# 5-HT2B-mediated serotonin activation in enterocytes suppresses colitis-associated cancer initiation and promotes cancer progression

**DOI:** 10.7150/thno.70762

**Published:** 2022-05-09

**Authors:** Liyuan Mao, Fang Xin, Jie Ren, Shuai Xu, Haixia Huang, Xu Zha, Xinxin Wen, Guoqing Gu, Guang Yang, Yuan Cheng, Chen Zhang, Wei Wang, Xicheng Liu

**Affiliations:** 1 Department of Physiology and Pathophysiology, School of Basic Medical Sciences, Capital Medical University, Beijing, China.; 2 Department of Immunology, School of Basic Medical Sciences, Capital Medical University, Beijing, China.; 3 Department of Neurobiology, School of Basic Medical Sciences, Capital Medical University, Beijing, China.; 4 Beijing Key Laboratory of Cancer Invasion and Metastasis Research, School of Basic Medical Sciences, Capital Medical University, Beijing, China.

**Keywords:** Serotonin, 5-HT2B, colitis-associated cancer, TGF-β

## Abstract

**Rationale:** Serotonin (5-hydroxytryptamine, 5-HT) is generally considered to be involved in colitis-associated cancer (CAC), but previous research has yielded inconsistent results regarding the effect of 5-HT on CAC. 5-HT2B is one of the receptors of 5-HT, and the receptor is expressed in intestinal epithelial cells (IECs). However, the functions of 5-HT2B in CAC remain unclear. Our work demonstrates the variable functions of 5-HT/5-HT2B signaling in the initiation and progression of CAC in mice.

**Methods:** We constructed two types of mutant mice homozygous knockout of *Htr2b*, the gene encoding 5-HT2B, in IECs (*Htr2b*^ΔIEC^ and *Htr2b*^ΔIEC-ER^) to study the role of 5-HT2B in AOM/DSS-induced CAC model. Inflammation was measured using the body weight, colon length, and colitis severity score, and by histologic analysis of colon tissues. Tumor severity was assessed by tumor quantity, load, and histologic analysis of colon tumor tissues.

**Results:** In *Htr2b*^ΔIEC^ mice, AOM/DSS induced an enhancement of colitis and tumor severity. This process was due to the inhibition of TGF-β/SMAD signaling pathway and activation of IL-6/STAT3 signaling pathway. IL-6 antibody treatment reversed the stimulating effect of *Htr2b* deletion on tumorigenesis. However, tumor severity decreased in *Htr2b*^ΔIEC-ER^ mice injected with tamoxifen on day 48 of AOM/DSS treatment. Knockout Akt1 eliminated the function of 5-HT in promoting tumor cells.

**Conclusion:** Our work elucidates 5-HT/5-HT2B/TGF-β signaling as a critical tumor suppressing axis during CAC initiation but as a promoter of cancer progression in the late-stage of CAC. Our findings provide a new understanding of the role of 5-HT in the initiation and progression of CAC, offering a new perspective on the long-standing debate on whether the 5-HT signal promotes or inhibits tumors.

## Introduction

Colorectal cancer (CRC) is the third most common malignant cancer in the world and the second-highest cause of cancer-related mortality 1. Colitis-associated cancer (CAC) is a CRC subtype closely related to early inflammation 2, which mainly occurs in patients with inflammatory bowel disease (IBD) 3, 4. Oncogenesis is regulated by cancer-related factors. These factors are usually classified as oncogenes and tumor suppressor genes, but more and more genes violate this classification. For example, transforming growth factor β (TGF-β) acts as a tumor suppressor in premalignant cells 5. Inactivating mutations of TGF-β can cause severe IBD 6, 7. However, in the late- stage of tumor progression, TGF-β acts as an oncogene to promote angiogenesis and induce epithelial-mesenchymal transformation (EMT), thereby promoting the proliferation and metastasis of cancer cells 8. Although the dual role of TGF-β in early versus advanced cancer has been known for decades 5, the factors triggering functional changes in TGF-β activity are still not fully clear.

Serotonin (5-hydroxytryptamine, 5-HT) is a paracrine signaling molecule in the peripheral nervous system that is mainly secreted by enterochromaffin (EC) cells in the intestinal mucosa, and the vast majority of 5-HT is located in the gut 9. 5-HT is generally thought to promote intestinal inflammation and CAC 10, 11. The severity of colitis is reduced in tryptophan hydroxylase 1 (TPH1, the key enzyme for the synthesis of 5-HT)-deficient mice 12. 5-HT promotes colorectal tumor growth and mediates tumor angiogenesis 13. However, others have found that fluoxetine, an inhibitor of 5-HT reuptake, increases the level of 5-HT in the colon, alleviates inflammation, and inhibits the development of CAC 14. These results suggest that 5-HT may play a dual regulatory role in colitis and CAC. Increasing evidence further shows that 5-HT activity may protect the colon from early CRC-related events 15, 16. The Arisha group showed that this protection is associated with a significant decrease in 5-HT levels, as indicated by parathion treatment, resulting in increased levels of free radicals, DNA damage, and apoptosis 16. These findings indicate that 5-HT has a complex but not fully understood function in the regulation of colorectal cancer.

The effects of 5-HT are mediated by its interactions with receptors, mainly in the 5-HTR family, which activates multiple signaling pathways to exert physiological functions 17. Many studies have confirmed that 5-HT can promote tumor cell proliferation by binding to 5-HT1 and 5-HT2 receptors in a variety of malignant tumors, such as CRC 18. However, several genetic studies support a tumor suppressor role for the 5-HT1B gene in the tumorigenesis of many tumors in humans 19, 20. Jiang and colleagues indicated that the absence of 5-HT2B in pancreatic cancer cells could inhibit the proliferation of xenografts 21, but Ebrahimkhani reported that blocking 5-HT2B could enhance the proliferation of hepatic parenchyma cells 22. Moreover, the 5-HT3 receptor antagonist tropisetron can also inhibit the development of CAC by attenuating inflammatory reactions in colitis 23. These findings show that the 5-HTR family plays an important role in the regulation of 5-HT in tumors.

In this study, we used a mouse model of AOM/DSS-induced CAC to study the function and mechanism by which 5-HT regulates intestinal tumors. We confirmed that 5-HT acts mainly via 5-HT2B receptors in the intestinal epithelium during AOM/DSS-induced colonic tumorigenesis. We then examined the impact of specific knockout of the *Htr2b* gene encoding 5-HT2B in intestinal epithelium at different stages of CAC, and further clarified the dual role of 5-HT in the initiation and development of CAC. Finally, we studied the molecular mechanisms by which 5-HT/5-HT2B signaling regulates colorectal tumors at different stages of CAC and elucidated the involvement of the TGF-β signaling pathway.

## Methods

### Experimental animals

Mice were bred and raised in a specific pathogen-free facility with access to standard animal chow and water. Statistical methods were not used to pre-determine the sample size, and all experiments were conducted on sex-matched mice at 8-10 weeks of age. *Htr2b^lox/lox^* mice were hybridized with *villin*-Cre and *villin*-CreER^T2^ mice to produce* Htr2b*^ΔIEC^ mice and *Htr2b*^ΔIEC-ER^ mice, respectively. *Htr2b* and *Stat3* double knockout (*Htr2b*^ΔIEC^; *Stat3*^ΔIEC^) mice were generated by hybridization of *Htr2b*^ΔIEC^ mice and *Stat3*^ΔIEC^ mice. Cre-negative littermates were used as a wild-type (WT) control. For *villin*-CreER^T2^ induction, mice were intraperitoneally injected with tamoxifen (Sigma-Aldrich, St. Louis, MO, T5648) dissolved in sunflower oil at a concentration of 2 mg per 20 g body weight for 5 consecutive days.

Inducible SMAD4-overexpressing mice (*Rosa26-lox-STOP-lox-COUP-Smad4*;* Smad4*^OE*/+*^) were generated by microinjection of in vitro-translated Cas9 mRNA, sgRNA, and a donor vector containing *Smad4* cDNA (depicted in Figure S4A) into C57BL/6 zygotes. The founders were verified by sequencing the PCR fragments.

### Induction of CAC or colitis

To induce CAC, mice (8-10 weeks old) were intraperitoneally injected with a single dose of azoxymethane (AOM, 10 mg/kg; Sigma-Aldrich, St Louis, MO, A5486). After 5 days, 2.5% dextran sodium sulfate (DSS, molecular weight 36-50 kDa; MP Biomedicals, Santa Ana, CA, 0216011080) was administered via the drinking water for 5 days, after which regular drinking water was supplied for 14 days. This DSS regimen was repeated for two additional cycles, and the mice were sacrificed 80 days after the AOM injection, except when indicated otherwise. Polyp counts and immunohistochemistry analyses were performed by investigators blinded to the treatments. The number of polyps was determined as the total number of polyps in a given mouse. Polyp load was identified as the sum of the diameters of all polyps in a given mouse 24.

For acute colitis and inflammation studies, mice (8-9 weeks old) were intraperitoneally injected with a single dose of AOM (10 mg/kg). After 5 days, 3% DSS was dissolved in the water fed to the mice for an additional 5 days, and the mice were sacrificed 15 days after the AOM injection. We administered either SB-204741 (3 mg/kg, i.p.; Sigma-Aldrich, S0693), compound-15 (30 mg/kg, oral; gift of Dr Yu Zhou, Beijing, China), fluoxetine (10 mg/kg, i.p.; Sigma-Aldrich, F132), or vehicle to age-matched C57BL/6 mice for the 5-HT2 antagonist or agonist studies. Body weights were recorded.

To study the influence of IL-6 signaling on CAC formation and growth, anti-human IL-6 antibody (20 mg/kg; Siltuximab, CNT0328) was administered intraperitoneally 3 times per week from day 0 of the CAC mouse model.

In all experiments, littermate controls were used to compare mice with the same genetic background. Animals that showed health problems unrelated to the study conditions were excluded from the analysis.

### Histology and immunohistochemistry

For histology, colons were removed from mice, gently rinsed with cold PBS, opened longitudinally, fixed as “Swiss rolls” in a 10% formalin solution (Sigma-Aldrich, St. Louis, MO, HT-501128) at room temperature overnight, and embedded in paraffin. Serial sectioning (5 µm) was performed, and every 40th section was stained with hematoxylin and eosin (H&E) or Alcian blue.

For immunohistochemical staining, paraffin-embedded colon sections were stained with antibodies against 5-HT2B (Abcam, ab194333, dilution 1:500), 5-HT (Sigma-Aldrich, S5545, dilution 1:4000), Ki-67 (Cell Signaling Technology (CST), 12202, dilution 1:400), cleaved caspase-3 (CST, 9661, dilution 1:300), phospho-STAT3 (Tyr705) (CST, 9145, dilution 1:400), STAT3 (CST, 4904, dilution 1:500), phospho-ERK (CST, 4370, dilution 1:400), p21 (CST, 2947, dilution 1:50), cyclin D1 (CST, 55506, dilution 1:250), phospho-Akt (Ser473) (CST, 4060, dilution 1:200), α-SMA (Abcam, Ab5694, dilution 1:200), and chromogranin A (Epitomics, 1782-1, dilution 1:200) overnight at 4℃. The secondary antibodies were horseradish peroxidase (HRP)-conjugated anti-rabbit IgG (CST, 7074, dilution 1:200) and anti-mouse IgG (CST, 7076, dilution 1:200), incubated at room temperature for 1 h, and purchased from CST.

### RNA extraction and quantitative real-time PCR (qPCR)

Tissues were homogenized in TRIzol (Life Technologies) using a rotor-stator homogenizer, and total RNA was extracted according to the manufacturer's instructions. The isolated RNA was digested with DNase I and purified using an RNeasy Mini kit (Qiagen, 74104). The purified RNA was reverse transcribed using the HiScript II 1st Strand cDNA Synthesis Kit (Vazyme, R211-02), and the cDNA template was mixed with ChamQ SYBR qPCR master mix reagents (Vazyme, Q331-02). Real-time PCR was performed using the ABI 7500 Fast Real-Time PCR System (Applied Biosystems). Relative quantities (Δ cycle threshold values) were obtained by normalizing against those of GAPDH. The primers used are listed in Table S1.

### Western blot analysis

Total protein was extracted from cultured cells or mouse intestinal epithelial cells for 30 min on ice by standard methods using RIPA lysis buffer (150 mM sodium chloride, 1.0% (vol/vol) Triton X 100, 0.5% sodium deoxycholate, 0.1% SDS, 50 mM Tris, pH 8.0) containing a protease inhibitor cocktail (Roche, Basel, Switzerland, 04693124001) and phosphatase inhibitor cocktail (Roche, Basel, Switzerland, 4906837001). The lysates were then centrifuged at 12000 rpm for 15 min at 4°C. The supernatant was collected, and the protein concentration was determined using a BCA protein assay kit (Thermo, Waltham, MA, 23227). Equal amounts of protein (15-25 µg) were separated by standard SDS-PAGE on an 8%-12% polyacrylamide separating gel with a 5% stacking gel before being transferred to PVDF membranes (Millipore, IPVH00010) by standard methods. The following primary antibodies were used: anti-phospho-STAT3 (Tyr705) (9145), anti-STAT3 (4904), anti-SMAD2 (5339), anti-phospho-SMAD2 (Ser465/467) (3108), anti-phospho-SMAD3 (Ser423/425) (9520), anti-SMAD3 (9523), anti-SMAD4 (38454), anti-phospho-ERK (4370), anti-ERK (9102), anti-phospho-Akt1 (Ser473) (9018), anti-Akt1 (75692), anti-phospho-Akt2 (Ser474) (8599), anti-Akt (2920) and anti-β-actin (3700) (all from CST MA, USA); and anti-5-HT2B (ab102707), anti-phospho-JunD (Ser255) (ab139180), and anti-JunD (ab134067) (all from Abcam, Cambridge, MA, USA). All primary antibodies were diluted 1:1000-2000. The HRP-linked secondary antibodies anti-mouse IgG (7076) and anti-rabbit IgG (7074) were purchased from CST and diluted 1:2000.

### Cell culture

CT26.WT cells were obtained from ATCC (Manassas, VA, CRL-2638) and maintained in RPMI 1640 medium (ATCC, 30-2001). The RPMI 1640 medium was supplemented with 10% fetal bovine serum (FBS, Gibco, Grand Island, NY), 100 U/mL penicillin and 100 μg/mL streptomycin (Life Technologies), except as indicated. Cells were grown at 37°C in a 5% CO_2_ incubator. The CT26.WT cell line was identified with short tandem repeat (STR) profiling by the ATCC. Upon receipt from ATCC, the cells were expanded and subsequently stored in liquid nitrogen. The stored vials were thawed for experiments and used in <2 months. All cell lines were confirmed to be negative for mycoplasma by ATCC.

### Sample preparation for HPLC

Solutions A (0.4 M perchloric acid) and B (20 mM potassium citrate, 300 mM dipotassium hydrogen phosphate, 2 mM EDTA·2Na) were prepared. The samples (50-60 mg) were homogenized in 150 μl of cold solution A (protected from light), incubated on ice for 1 h, and then centrifuged for 20 min at 12000 rpm at 4°C. The clear supernatant was transferred to a new clear tube, and then solution B (half the volume of the transferred supernatant) was added and vortexed. The samples were further incubated on ice for another 1 h, and then centrifuged for 20 min at 12000 rpm at 4°C. The clear supernatant was transferred to a new clear tube and stored at -80°C. HPLC of the samples was performed by the Central Laboratory of Capital Medical University. A Waters e2695/2165 mass spectrometer was used.

### Generation of CRISPR-Cas9 knockout cell lines

CRISPR-Cas9 knockout lentivirus targeting Akt1 and the corresponding control vector were purchased from GeneChem Company (Shanghai, China). The sgRNA sequence for Akt1 was as follows: 5′-CATTGAGCGCACCTTCCATG-3′. CT26.WT cells were infected with lentivirus, and cells with stable expression of Cas9 and sgRNA were screened with 2.5 μg/mL puromycin (InvivoGen, San Diego, CA, ant-pr-1) for 72 h. Single clones were selected using a monoclonal technique. Briefly, cells were trypsinized and diluted to a concentration of 10 cells/mL, and 100 μL cell suspension was added to each well in a 96-well plate (i.e., one cell per well). The cells were then cultured in a humidified incubator at 37°C with 5% CO_2_ for 10-14 days until a cell colony formed. The cells were subjected to trypsin digestion, transferred to a 24-well plate, and cultured for 7-10 days. When the cells reached 70%-80% confluence, they were passaged into a 6-well plate. The final step included trypsin digestion and extraction of DNA and protein for further validation. The homogeneity of the clones was verified by screening using a gene knockout and mutation detection kit (GeneChem Company, MB001-1004), and the candidate knockout clones were verified by sequencing PCR fragments and western blotting.

### Transwell assay

CT26.WT cells with Akt1 knockout (3×10^4^) in serum-free medium containing 5 μM serotonin (Sigma-Aldrich, H9523) or PBS were plated in the upper chamber of 8.0-*μm* pore-size inserts (Corning, Bedford, MA, 3422) coated with Matrigel (Corning, 354234) for invasion assays or left uncoated for migration assays. A medium containing 10% FBS as a chemoattractant and either 5 μM serotonin or PBS was added to the lower chamber. After the cells were incubated at 37°C for 48 h for the migration assay or 72 h for the invasion assay, the non-migrating or non-invasive cells were removed from the upper surface of the Transwell membrane using cotton swabs. The migrated or invaded cells were fixed with 4% paraformaldehyde and stained with 0.1% crystal violet in methanol, and the field count was randomly selected under a light microscope.

### Wound healing assay

The cells were grown as a confluent monolayer in a six-well culture dish. The cell monolayers were scratched using a sterile p200 pipette tip to create a wound, and then washed with PBS to remove cell debris. The cells were incubated in medium supplemented with 0.5% FBS and either 5 μM serotonin or PBS. Cell migration was monitored under an inverted microscope equipped with a camera. The wound distance (width) at different time points was measured. The open wound (%) was calculated as the wound distance/AWD0×100, where AWD0 is the average wound distance at 0 h.

### CCK-8 assay

Cells were seeded at a density of 3000 per well in 96-well plates and cultured for 8, 24, 48, or 72 h in 100 μL medium containing 10% FBS. Next, 10 μL CCK-8 solution (NCM Biotech, C6005) was added per well, and the cells were cultured for another 2 h at 37°C. The number of viable cells was evaluated by measuring the absorbance at 450 nm using a SynergyH1 microplate reader.

### Tumor allograft assay

CT26.WT cells transduced with the Akt1 knockout vector or corresponding control vector (5×10^4^ cells per mouse) were subcutaneously injected into 6-week-old female nude mice. A week later, each allograft tumor-bearing mouse was randomly assigned to one of two groups (n = 7) and intraperitoneally injected with 100 μL PBS or serotonin chloride (10 mg/kg) (Sigma-Aldrich, H9523) three times a week for two weeks. Tumor volume was calculated based on the tumor width (a) and length (b) measured with Vernier calipers (TV = (a2b)/2); volumes were recorded every day from the beginning of PBS or serotonin chloride treatment. Three weeks after the cell injection, the mice were euthanized, and the weights of the tumors in these groups were measured.

For the Akt antagonist assay, CT26.WT cells were subcutaneously injected into 6-week-old female nude mice. A week later, each allograft tumor-bearing mouse was randomly assigned to one of three groups (n = 9) and intraperitoneally injected with serotonin chloride (10 mg/kg) (Sigma-Aldrich, H9523) and/or GSK690693 (30 mg/kg) (Selleck, S1113), or PBS three times a week for two weeks.

### Human colon adenocarcinoma tissue microarray analysis

A series of tissue microarrays (Shanghai Outdo Biotech Co., Ltd.) consisting of matched pairs of primary human colon adenocarcinoma samples and adjacent normal tissues from 271 patients with survival, and primary human colon adenocarcinoma samples from 292 patients was used. Protein levels in the tissues were determined by staining sections of the tissue microarray with antibodies targeting human 5-HT2B (ab140495, 1:500, Abcam), human p21 (CST, 2947, dilution 1:50), and cyclin D1 (CST, 55506, dilution 1:250). Positive staining was scored as follows: high, large area of staining; moderate, staining of multiple smaller areas; low, staining of a few scattered cells; and no, no positive cells.

Another set of CRC samples (20 patients) was purchased from the Shanghai Outdo Biotech Company. The specimens were diagnosed using immunohistochemistry techniques. This experiment was approved by the Ethics Committee of Capital Medical University (approval no. AEEI-2019-152).

### Statistics and reproducibility

All results are presented as the mean ± SEM. The statistical details, including the definitions and value of n (e.g., number of experimental replicates, micrographs, cells, animals, etc.), are provided in the figures and corresponding figure legends. Statistical significance was determined by a two-tailed unpaired Student's t-test, One-way ANOVA, Two-way ANOVA, and log-rank test using GraphPad Prism 9 (GraphPad Software) software. P values less than 0.05 were considered statistically significant.

## Results

### 5-HT2B deficiency in IECs exacerbates CAC tumorigenesis

5-HT2B was expressed in the IECs (Figure S1A). To study the function of this receptor in the intestinal tract, we generated mice containing floxed *Htr2b* alleles and a transgene expressing Cre recombinase under the control of the intestine-specific *villin* gene promoter (*villin*-Cre) (hereafter referred to as *Htr2b*^ΔIEC^ mice). The efficiency of 5-HT2B knockout was confirmed by immunohistochemical analysis of IECs (Figure S1A) and immunoblot analysis of isolated colonic epithelial cells (Figure S1B). IEC-specific 5-HT2B depletion had no significant effect on epithelial homeostasis in the colon (Figures S1C and D), and the baseline rates of cell proliferation and apoptosis in the colonic epithelium measured by Ki-67 (a marker of cellular proliferation) and cleaved caspase-3 (a marker of apoptosis), respectively, were also indistinguishable between wild-type (WT) mice and *Htr2b*^ΔIEC^ mice (Figures S1E and F). Further, *Htr2b*^ΔIEC^ mice were fertile and healthy, with no obvious phenotypic disturbances (Figures S1C-F). These data suggest that 5-HT2B is largely dispensable with respect to intestinal epithelium development and homeostasis.

**Figure 1:**
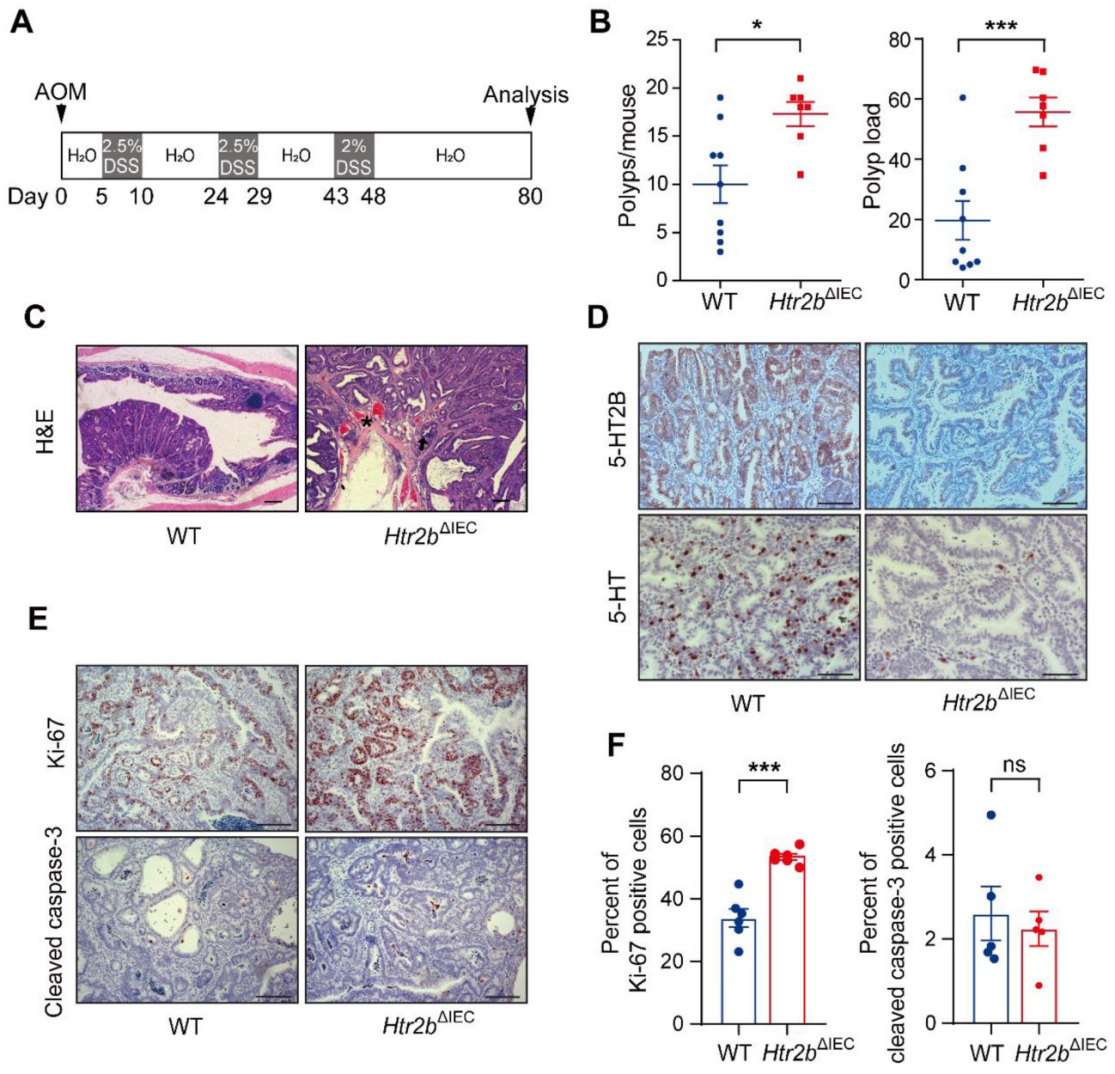
** The deficiency of 5-HT2B promotes the occurrence of CAC.** (**A**) Schematic overview of the AOM/DSS model of CAC in mice. (**B**) The colonic polyp multiplicity and polyp load in WT (n = 9) and *Htr2b*^ΔIEC^ (n = 7) mice following the induction of CAC using AOM/DSS. Each data point represents a single mouse. (**C**) Hematoxylin-eosin (H&E) staining of representative colonic sections from WT and *Htr2b*^ΔIEC^ mice treated with AOM/DSS. Arrow: aggressive adenomas with invasion; asterisk: muscularis mucosa and angiogenesis. (**D**) Typical immunohistochemical analysis of 5-HT2B and 5-HT in colonic polyp tissues from WT and *Htr2b*^ΔIEC^ mice. (**E**) Immunohistochemical analysis of Ki-67 and cleaved caspase-3 in colonic polyp tissues of WT and *Htr2b*^ΔIEC^ mice. (**F**) The percentage of Ki-67 (n = 6) and cleaved caspase-3 (n = 5) positive cells (positive epithelial cells/total epithelial cells) in colonic polyp tissues of WT and *Htr2b*^ΔIEC^ AOM/DSS-treated mice. Error bars represent the mean ± SEM. Scale bars, 100 *μ*m. ns: not significant, ^*^P < 0.05, ^***^P < 0.001; unpaired Student's t-test (B, F).

As inhibition of 5-HT signaling was shown to hinder the growth of a CRC cell line in a xenograft tumor model 25, we sought to examine the impact of inhibition of 5-HT signaling via *Htr2b* knockout on the tumorigenesis of CAC and determine whether 5-HT acts as a tumor promoter in this disease. To investigate the role of 5-HT /5-HT2B in CAC initiation, WT and *Htr2b*^ΔIEC^ mice were injected with the carcinogen azoxymethane (AOM) and then treated with three rounds of oral administration of the luminal toxin dextran sodium sulfate (DSS) to induce CAC (Figure 1A). After AOM/DSS treatment (day 80), the colonic polyp multiplicity and polyp load of *Htr2b*^ΔIEC^ mice were significantly increased compared with those of WT mice (Figure 1B). Colonic adenomas observed in WT mice generally displayed only low-grade dysplasia, but the lesions in *Htr2b*^ΔIEC^ mice presented as more aggressive adenomas with invasion of tumor cells below the muscularis mucosa and angiogenesis (Figure 1C). Immunohistochemical analysis showed that 5-HT2B was successfully knocked down in the colonic epithelium of *Htr2b*^ΔIEC^ mice. Compared with the 5-HT levels in the colonic epithelium of WT mice, those of *Htr2b*^ΔIEC^ mice were significantly reduced (Figure 1D). To exclude the possibility that the reduction in 5-HT levels was not due to a decrease in synthesis, we performed immunohistochemical staining for chromogranin A (a marker of EC cells) and 5-HT in colonic epithelial tumor tissues from WT mice. The results showed that only a few EC cells were present in the tissues, while 5-HT staining had a high positive rate (Figure S2A). We also detected 5-HT levels in the colon tissues and IECs of WT and *Htr2b*^ΔIEC^ mice. We found no significant difference in 5-HT levels in colon tissues between WT and *Htr2b*^ΔIEC^ mice, but there was a significant decrease in the IECs of *Htr2b*^ΔIEC^ mice (Figure S2B). These results indicate that there was no change in 5-HT synthesis. The reduction in 5-HT was due to the fact that 5-HT could not bind to 5-HT2B when 5-HT2B was knocked out in IECs, leading to a significant decrease in the localization of 5-HT in IECs. Immunohistochemical staining for Ki-67 revealed that the proliferation of polyp cells in *Htr2b*^ΔIEC^ mice was significantly increased compared with that in WT mice (Figures 1E and F). We also analyzed apoptosis (cleaved caspase-3) in these polyps and found no obvious difference between the WT and *Htr2b*^ΔIEC^ mice (Figures 1E and F). These data indicate that the effects of 5-HT in dysplasia are mainly due to signaling through 5-HT2B and suggest that deletion of *Htr2b* blocks the antineoplastic activity of 5-HT.

To rule out any potential confounding effect of changes in development with *Htr2b* knockout, we generated mice containing floxed *Htr2b* alleles and the *villin*-Cre-ER^T2^ driver (hereafter referred to as *Htr2b*^ΔIEC-ER^ mice), which allows controlled gene ablation in IECs upon tamoxifen treatment; we administered tamoxifen 5 times during the first DSS treatment window in the AOM/DSS treatment regime (Figure S2C). Similar to *Htr2b*^ΔIEC^ mice (Figure 1B), *Htr2b*^ΔIEC-ER^ mice had significantly more polyps and a higher polyp load than did WT mice (Figure S2D). The cell proliferation in polyps was significantly increased in *Htr2b*^ΔIEC-ER^ mice, but apoptosis was not different between *Htr2b*^ΔIEC-ER^ and WT mice (Figures S2E and F). Taken together, our findings indicate that a lack of 5-HT2B signaling in IECs promotes the initiation of colorectal neoplasia in CAC.

### *Htr2b*^ΔIEC^ mice are more susceptible to DSS-induced colitis and epithelial damage than WT mice

**Figure 2:**
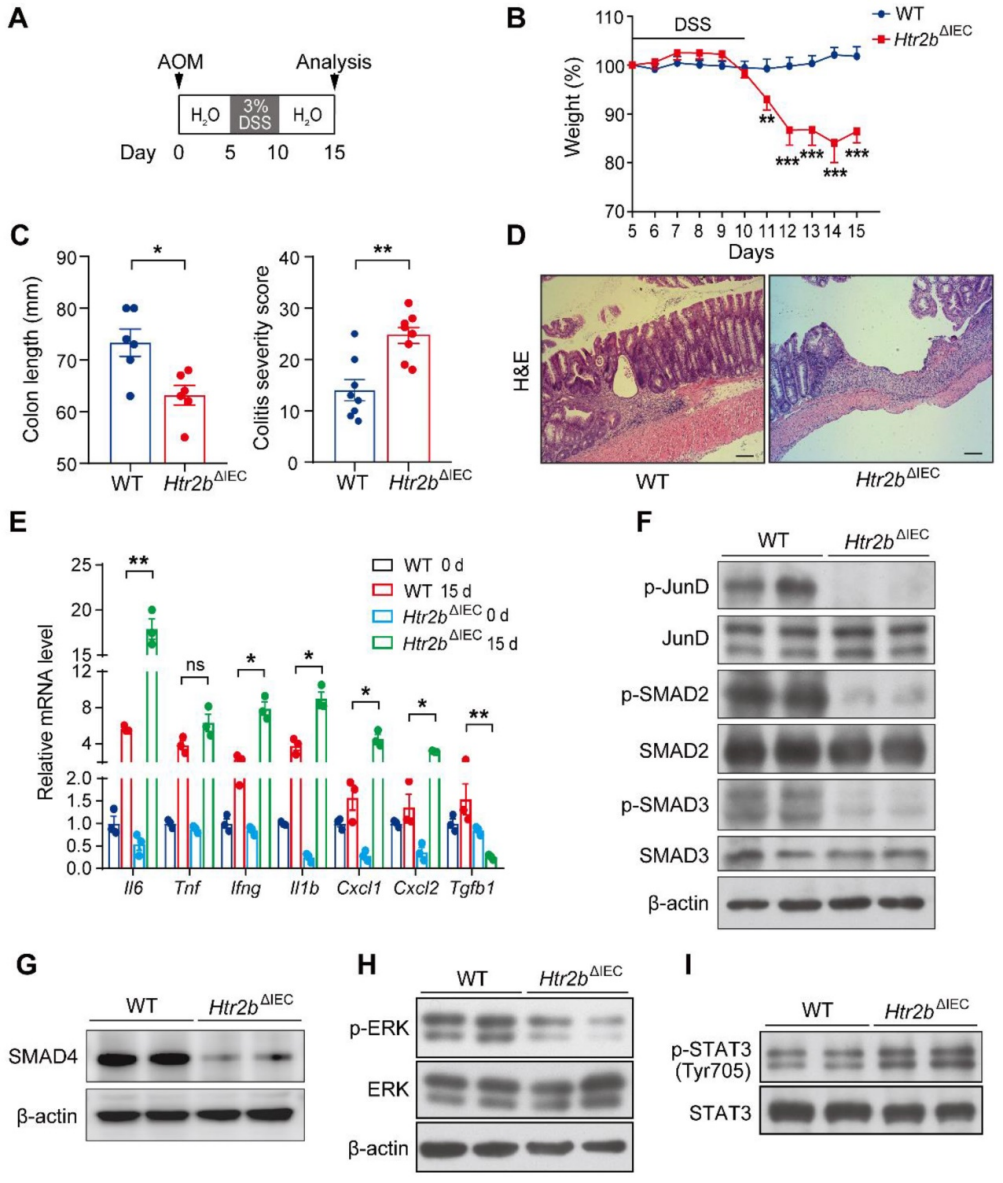
** Deletion of 5-HT2B inhibits the TGF-β signaling pathway and enhances the inflammatory response.** (**A**) Schematic overview of the AOM/DSS model of acute colitis in mice. (**B**) Body weight of WT (n = 8) and *Htr2b*^ΔIEC^ (n = 8) mice during AOM/DSS treatment. (**C**) The colon length and colitis severity score of WT and *Htr2b*^ΔIEC^ mice (n = 6-8) with acute colitis. (**D**) H&E staining of colon sections from WT and *Htr2b*^ΔIEC^ mice with acute colitis on day 15. (**E**) Proinflammatory cytokine mRNA levels (n = 3) in whole colonic mucosa specimens from WT and *Htr2b*^ΔIEC^ mice. (**F**) Levels of total protein and phosphorylated JunD, SMAD2, and SMAD3 in the colonic epithelium of WT and *Htr2b*^ΔIEC^ mice. (**G**) SMAD4 protein levels in the colonic epithelium of WT and *Htr2b*^ΔIEC^ mice. (**H**) Levels of total protein and phosphorylated ERK in the colonic epithelium of WT and *Htr2b*^ΔIEC^ mice. (**I**) Levels of total protein and phosphorylated STAT3 in the colonic epithelium of WT and *Htr2b*^ΔIEC^ mice. Scale bars, 100 *μ*m. Error bars represent the mean ± SEM. ns: not significant, ^*^P < 0.05, ^**^P < 0.01, ^***^P < 0.001; Two-way ANOVA (B), unpaired Student's t-test (C) and One-way ANOVA (E).

It is well known that inflammation plays a key role in tumorigenesis 26. We speculated that the increased polyp multiplicity and load in *Htr2b*^ΔIEC^ mice could be linked to increased intestinal inflammation. To investigate the role of 5-HT2B in the inflammation of CAC, we induced acute colitis by administering 3% DSS in the drinking water for the *Htr2b*^ΔIEC^ and WT mice for 5 days beginning on day 5 after AOM injection (Figure 2A). Upon the first cycle of DSS treatment, *Htr2b*^ΔIEC^ mice showed more body weight loss, more extensive colon shortening, and higher colitis scores than WT mice (Figures 2B and C), indicating that *Htr2b*^ΔIEC^ mice probably had enhanced inflammation and increased colonic epithelial damage. Histological analysis on day 15 confirmed that colons from *Htr2b*^ΔIEC^ mice exhibited moderate to severe inflammation, with many areas presenting complete crypt loss and erosion, compared to the partially conserved epithelial structures observed in WT mice (Figure 2D). As epithelial apoptosis is one of the mechanisms of DSS-induced intestinal inflammation and colitis 27, we found that treatment with DSS induced more apoptosis in the IECs of* Htr2b*^ΔIEC^ mice than in WT mice through immunohistochemical staining for cleaved caspase-3 (Figure S3A). In response to DSS-induced damage, the intestinal epithelium also initiates repair and regeneration by increasing cell proliferation 28. Therefore, at day 15 of the AOM/DSS treatment, we detected Ki-67 in the colonic epithelium and found no significant increase in the number of positive cells in *Htr2b*^ΔIEC^ mice compared to WT mice (Figures S3A and B). These results indicate that the lack of 5-HT signaling through 5-HT2B increases IEC apoptosis and attenuates IEC resistance to DSS-induced colitis, with potential effects on tissue regeneration.

Body weight loss is one of the indicators of the severity of DSS-induced colitis 29. We treated AOM/DSS colitis model C57BL/6 mice with the 5-HT2B antagonists SB-204741 and Compound-15 and found that the changes in body weight (Figures S3C and D) corresponded to those observed with the *Htr2b*^ΔIEC^ colitis mouse model (Figure 2B). To eliminate the effect of AOM on early acute inflammation, we provided mice water containing 3% DSS for 5 days and monitored weight changes. The results showed that the weight loss in *Htr2b*^ΔIEC^ mice was greater than that in the WT mice (Figure S3E). Furthermore, fluoxetine, a 5-HT reuptake inhibitor that selectively inhibits the serotonin transporter (SERT) and thus prolongates and increases the action of 5-HT, was also used in DSS-treated C57BL/6 mice. The results showed that the body weight of the fluoxetine-treated mice was significantly higher than that of the control mice (Figure S3F). These results further suggest that 5-HT inhibits the inflammatory response in CAC, and this effect is mediated by 5-HT2B.

Consistent with this extensive hyperinflammatory response phenotype (Figure 2D), the results of qPCR analysis of colons collected at day 15 showed that the expression of *Il6*, *Ifng*,* Il1b*, *Cxcl1*, and* Cxcl2* genes encoding the proinflammatory molecules IL-6, IFNγ, IL-1β, the mouse chemokine (C-X-C motif) ligand homologs KC and MIP-2 was significantly increased in *Htr2b*^ΔIEC^ mice (Figure 2E). These data showed that increased epithelial damage was accompanied by increased inflammation. Therefore, the increased polyp multiplicity and polyp load in *Htr2b*^ΔIEC^ mice could be due to an elevated intestinal inflammatory response.

Notably, the expression of the *Tgfb1* gene encoding the transforming growth factor β1 (TGF-β1), which is closely associated with inflammation, was significantly reduced in *Htr2b*^ΔIEC^ mice (Figure 2E). Previously, the TGF-β signaling pathway was proven to be an important signaling pathway in the inhibition of CAC tumorigenesis 7, and the TGF-β/SMAD pathway is also an important tumor suppressor pathway in several tumors 8, 30. 5-HT/5-HT2B signaling affects the TGF-β1 signaling pathway by regulating the phosphorylation of mitogen-activated protein kinase 1 (ERK) and the transcription factor JunD in hepatic stellate cells, which negatively regulates hepatocyte regeneration 22. We performed western blot analysis, which indicated that the levels of SMAD4 and phosphorylated JunD, ERK, SMAD2, and SMAD3 were significantly decreased in the colonic epithelium of *Htr2b*^ΔIEC^ mice 15 days after initiation of the AOM/DSS regimen (Figures 2F-H). At 80 days after AOM/DSS treatment, immunohistochemical analysis revealed that the levels of phosphorylated ERK, SMAD2, and SMAD3 in the colonic epithelium of *Htr2b*^ΔIEC^ mice were still lower than those in that of WT mice (Figures S3G and H).

As previous studies have shown that TGF-β inhibits CAC by inhibiting the IL-6/STAT3 signaling pathway 7, 31, we also detected the phosphorylation of STAT3 and found that its levels were increased in the colonic epithelium of *Htr2b*^ΔIEC^ mice (Figure 2I). At 80 days after the initial AOM/DSS treatment, the level of phosphorylated STAT3 in the colonic epithelium was still higher in *Htr2b*^ΔIEC^ mice than in the WT mice (Figure S3I). Taken together, these data suggest that the loss of 5-HT/5-HT2B signaling inhibits the TGF-β signaling pathway, enhances the intestinal inflammatory response, and aggravates damage to IECs, thus promoting the initiation of colorectal neoplasia in CAC.

### IEC-specific overexpression of *Smad4* alleviates CAC tumorigenesis in 5-HT2B-deficient mice

**Figure 3:**
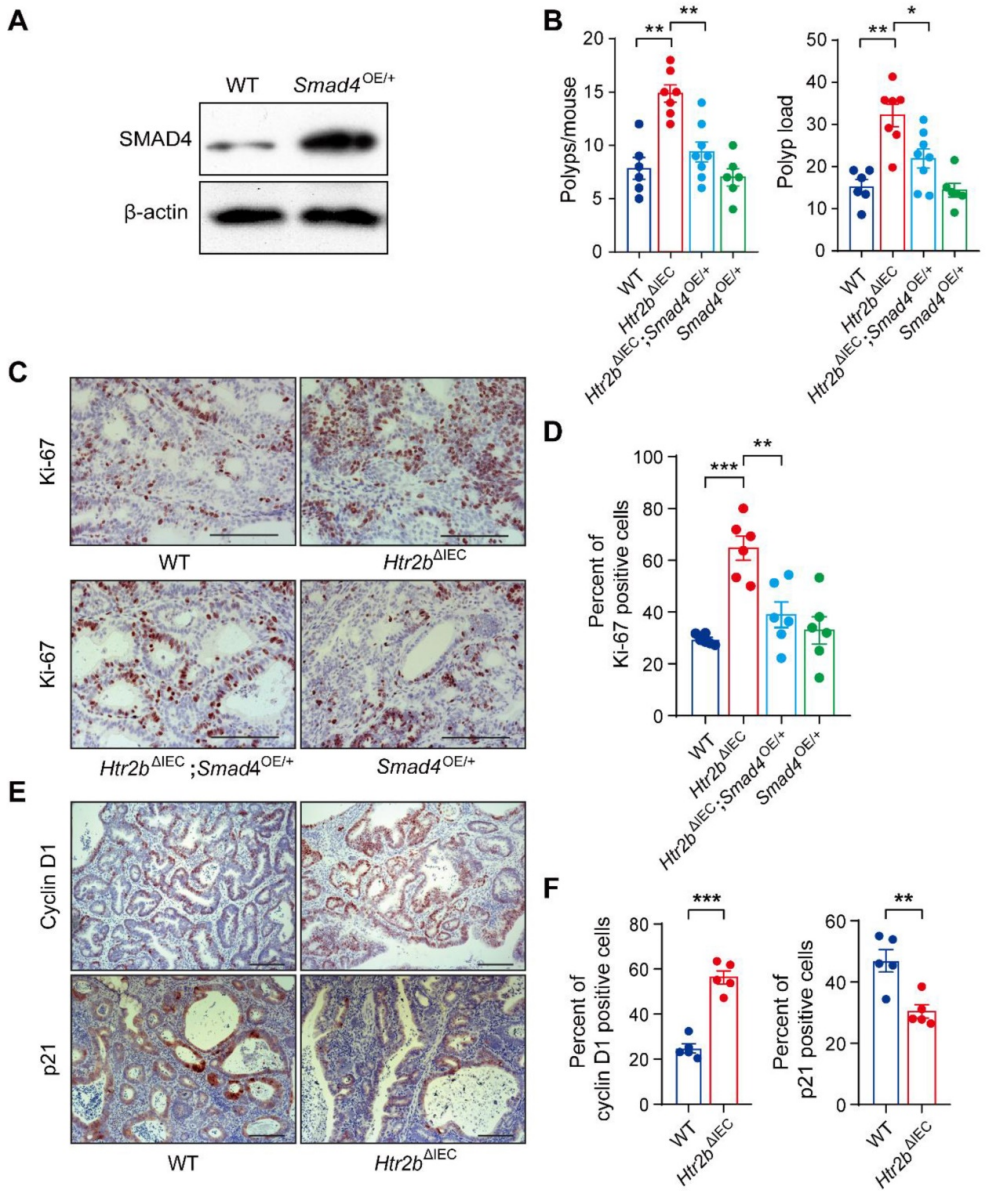
** Overexpression of *Smad4* alleviates CAC and regulates the proliferation of colonic epithelial cells in *Htr2b*^ΔIEC^ mice.** (**A**) The expression of SMAD4 in the colonic epithelium of WT and *Smad4*^OE/+^ mice. (**B**) The colonic polyp multiplicity and polyp load in WT, *Htr2b*^ΔIEC^, *Htr2b*^ΔIEC^;* Smad4*^OE/+^ and *Smad4*^OE/+^ mice treated with AOM/DSS (n = 6-8). (**C**) Immunohistochemical analysis of Ki-67 in the colonic polyp tissues of WT, *Htr2b*^ΔIEC^, *Htr2b*^ΔIEC^;* Smad4*^OE/+^ and *Smad4*^OE/+^ mice. (**D**) The percentage of Ki-67-positive cells in colonic polyp tissues of WT, *Htr2b*^ΔIEC^, *Htr2b*^ΔIEC^;* Smad4*^OE/+^ and *Smad4*^OE/+^ mice (n = 6). (**E**) Immunohistochemical analysis of cyclin D1 and p21 in colonic polyp tissues of WT and *Htr2b*^ΔIEC^ mice. (**F**) Cyclin D1- and p21-positive cell percentages in the colonic polyp tissues of WT and *Htr2b*^ΔIEC^ mice (n = 5). Scale bars, 100 *μ*m. Error bars represent the mean ± SEM. ^*^P < 0.05, ^**^P < 0.01, ^***^P < 0.001; One-way ANOVA (B, D) and unpaired Student's t-test (F).

Given that SMAD4 protein levels and phosphorylated levels of SMAD2 and SMAD3 were significantly downregulated in the colonic epithelium of *Htr2b*^ΔIEC^ mice treated with AOM/DSS (Figures 2F, G, and S3G), we sought to determine whether increasing TGF-β signaling would interfere with CAC tumorigenesis. Considering that the activation of TGF-β/SMAD pathway depends on elevated SMAD4 expression 32, 33, we generated a mouse line with inducible *Smad4* overexpression (*Smad4*^OE*/+*^) (Figure S4A) that allows constitutive SMAD4 expression in the intestinal epithelium. Western blot analysis showed that SMAD4 was overexpressed in the intestinal epithelium of *Smad4*^OE*/+*^ mice (Figure 3A). Mice with *Htr2b* knockout and *Smad4* overexpression (*Htr2b*^ΔIEC^; *Smad4*^OE*/+*^) were generated by crossing *Htr2b*^ΔIEC^ mice and *Smad4*^OE*/+*^ mice. We first assessed the effect of SMAD4 overexpression on colitis and found that the body weight of* Htr2b*^ΔIEC^; *Smad4*^OE*/+*^ mice recovered compared with *Htr2b*^ΔIEC^ mice (Figure S4B). Histological analysis on day 15 after AOM/DSS treatment confirmed a decrease in colonic epithelial damage of* Htr2b*^ΔIEC^; *Smad4*^OE*/+*^ mice compared with *Htr2b*^ΔIEC^ mice (Figure S4C). These results confirmed that SMAD4 overexpression attenuates inflammation induced by 5-HT2B deletion. We then assessed CAC and found that after AOM/DSS treatment, the polyp multiplicity and load of *Htr2b*^ΔIEC^ mice were greater than those of WT mice (Figure 3B), which was consistent with previous experimental results (Figure 1B). However, compared with* Htr2b*^ΔIEC^ mice, *Htr2b*^ΔIEC^; *Smad4*^OE*/+*^ mice showed a significantly decreased polyp multiplicity and load (Figure 3B). Immunohistochemical analysis of Ki-67 revealed decreased proliferation of colonic epithelial cells in *Htr2b*^ΔIEC^; *Smad4*^OE*/+*^ mice compared with *Htr2b*^ΔIEC^ mice (Figures 3C and D). The above experimental results indicate that SMAD4 overexpression in the intestinal epithelium is sufficient to overcome the CAC tumorigenesis induced by 5-HT2B deletion.

Cyclin D1 and p21 are both downstream targets of TGF-β signaling pathway 34. Overexpression of cyclin D1 is generally detected in different types of malignant tumors 35, 36, whereas p21, as a cell cycle inhibitor and an antiproliferative effector in normal cells, is dysregulated in some cancers 37. Therefore, we investigated p21 and cyclin D1 in CAC and found that cyclin D1 was upregulated, while p21 was downregulated in *Htr2b*^ΔIEC^ mice compared with WT mice (Figure 3E and F). These results suggested that 5-HT/5-HT2B inhibits the initiation of CAC through TGF-β/SMAD signaling pathway.

### Ablation of 5-HT2B exacerbates CAC tumorigenesis by activating the IL-6/STAT3 signaling pathway

**Figure 4:**
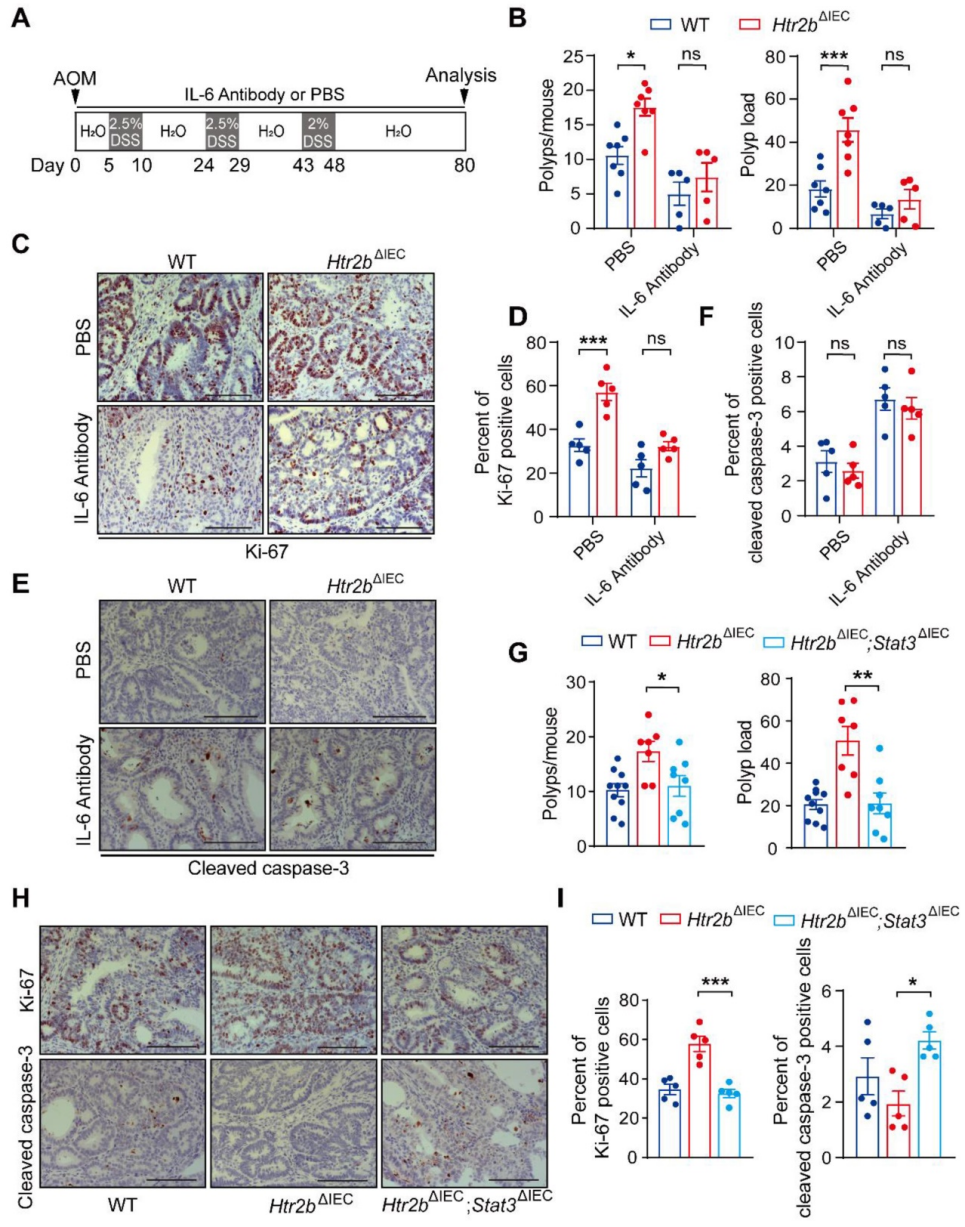
** Inhibition of IL-6 alleviates CAC in *Htr2b*^ΔIEC^ mice.** (**A**) A schematic overview of the AOM/DSS model of CAC with IL-6 antibody or PBS treatment in mice. (**B**) The colonic polyp multiplicity and polyp load of AOM/DSS-treated WT and *Htr2b*^ΔIEC^ mice (n = 5-7) with IL-6 antibody or PBS treatment. (**C**) Immunohistochemical analysis of Ki-67 in colonic polyp tissues from WT and *Htr2b*^ΔIEC^ mice treated with PBS or IL-6 antibody. (**D**) The percentage of Ki-67 positive cells in colonic polyp tissues of WT and *Htr2b*^ΔIEC^ mice treated with PBS or IL-6 antibody (n = 5). (**E**) Immunohistochemical staining for cleaved caspase-3 in colonic polyp tissues of WT and *Htr2b*^ΔIEC^ mice treated with PBS or an IL-6 antibody. (**F**) The percentage of cleaved caspase-3 positive cells in colonic polyp tissues of WT and *Htr2b*^ΔIEC^ mice treated with PBS or an IL-6 antibody (n = 5). (**G**) The colonic polyp multiplicity and polyp load in WT, *Htr2b*^ΔIEC^ and *Htr2b*^ΔIEC^; *Stat3*^ΔIEC^ (double-knockout of* Stat3* and *Htr2b* genes) mice treated with AOM and DSS (n = 7-10). (**H**) Immunohistochemical analysis of Ki-67 and cleaved caspase-3 in colonic polyp tissues of WT, *Htr2b*^ΔIEC^ and *Htr2b*^ΔIEC^; *Stat3*^ΔIEC^ mice treated with AOM and DSS. (**I**) The percentage of Ki-67 positive cells in the colonic epithelium of WT, *Htr2b*^ΔIEC^ and *Htr2b*^ΔIEC^; *Stat3*^ΔIEC^ mice (n = 5) treated with AOM and DSS. Scale bars, 100 *μ*m. Error bars represent the mean ± SEM. ns: not significant, ^*^P < 0.05, ^**^P < 0.01, ^***^P < 0.001; Two-way ANOVA (B, D, F) and One-way ANOVA (G, I).

TGF-β reduces colitis-associated tumorigenesis by inhibiting the IL-6/STAT3 signaling pathway^7^, ^31^, based on which, we hypothesized that the deficiency of 5-HT2B promotes the tumorigenesis of CAC through the attenuation of the inhibitory effect of TGF-β on IL-6/STAT3. To prove this hypothesis, we used IL-6 antibody to systematically block the effect of IL-6, and observed the effects on colitis and CAC in mice. For acute colitis, the body weight of WT mice and *Htr2b*^ΔIEC^ mice recovered after IL-6 antibody treatment compared with phosphate-buffered saline (PBS), and there was no significant difference between WT mice and *Htr2b*^ΔIEC^ mice after IL-6 antibody treatment (Figure S5A). Histological analysis on day 15 after AOM/DSS treatment confirmed that the colonic epithelial damage of WT and* Htr2b*^ΔIEC^ mice decreased after IL-6 antibody treatment, and there was no obvious difference between the two groups (Figure S5B). These results confirmed that IL-6 antibody treatment attenuated inflammation induced by 5-HT2B deletion. We then treated WT and *Htr2b*^ΔIEC^ CAC model mice with an IL-6 antibody or phosphate-buffered saline (PBS) (Figure 4A). In the PBS treatment group, the multiplicity and load of polyps in *Htr2b*^ΔIEC^ mice were significantly increased compared with WT mice, while there was no significant difference in the IL-6 antibody treatment group (Figure 4B). Immunohistochemical staining for Ki-67 revealed that the proliferation of colonic tumor enterocytes in *Htr2b*^ΔIEC^ mice treated with PBS was significantly increased compared with WT mice, while there was no significant difference in IL-6 antibody treatment (Figures 4C and D). There was no significant difference in cleaved caspase-3 expression between the WT and *Htr2b*^ΔIEC^ mice treated with IL-6 antibody (Figures 4E and F). These results suggest that IL-6 antibody treatment can reverse the promoting effect of 5-HT2B deletion on the tumorigenesis and development of CAC. 5-HT/5-HT2B regulates CAC may through the IL-6 signaling pathway.

We also found that the level of p-STAT3 decreased in the colonic polyp tissues of WT and *Htr2b*^ΔIEC^ mice after IL-6 antibody treatment, but there was no significant difference between the two groups (Figures S5C and D). This result indicates that STAT3 is involved in this pathway. To examine the function of STAT3 in this pathway, we generated mice containing floxed *Stat3* and *Htr2b* alleles and the *villin*-Cre promoter (hereafter referred to as *Htr2b*^ΔIEC^; *Stat3*^ΔIEC^ mice). We confirmed the absence of STAT3 expression in the IECs of *Htr2b*^ΔIEC^; *Stat3*^ΔIEC^ mice by immunoblot analysis of isolated IECs (Figure S5E) and immunohistochemical analysis of IECs (Figure S5F). After AOM/DSS treatment, the polyp multiplicity and load of *Htr2b*^ΔIEC^ mice were increased compared with those of WT mice, but double knockout of *Stat3* and *Htr2b* attenuated these increases to levels consistent with those of WT mice (Figure 4G). Correspondingly, immunohistochemical analysis of Ki-67 expression revealed that the proliferation in the colonic epithelium of *Htr2b*^ΔIEC^; *Stat3*^ΔIEC^ mice was lower than that of *Htr2b*^ΔIEC^ mice and more similar to that of WT mice (Figures 4H and I), and immunohistochemical staining for cleaved caspase-3 showed that epithelial apoptosis was moderately increased in *Htr2b*^ΔIEC^; *Stat3*^ΔIEC^ mice when compared with *Htr2b*^ΔIEC^ mice (Figures 4H and I). The above experimental results indicate that the tumorigenic role of 5-HT2B deficiency is abrogated by inhibiting the IL-6/STAT3 signaling axis.

### Knockout of *Htr2b* in the late-stage of CAC inhibits the development of tumors

**Figure 5:**
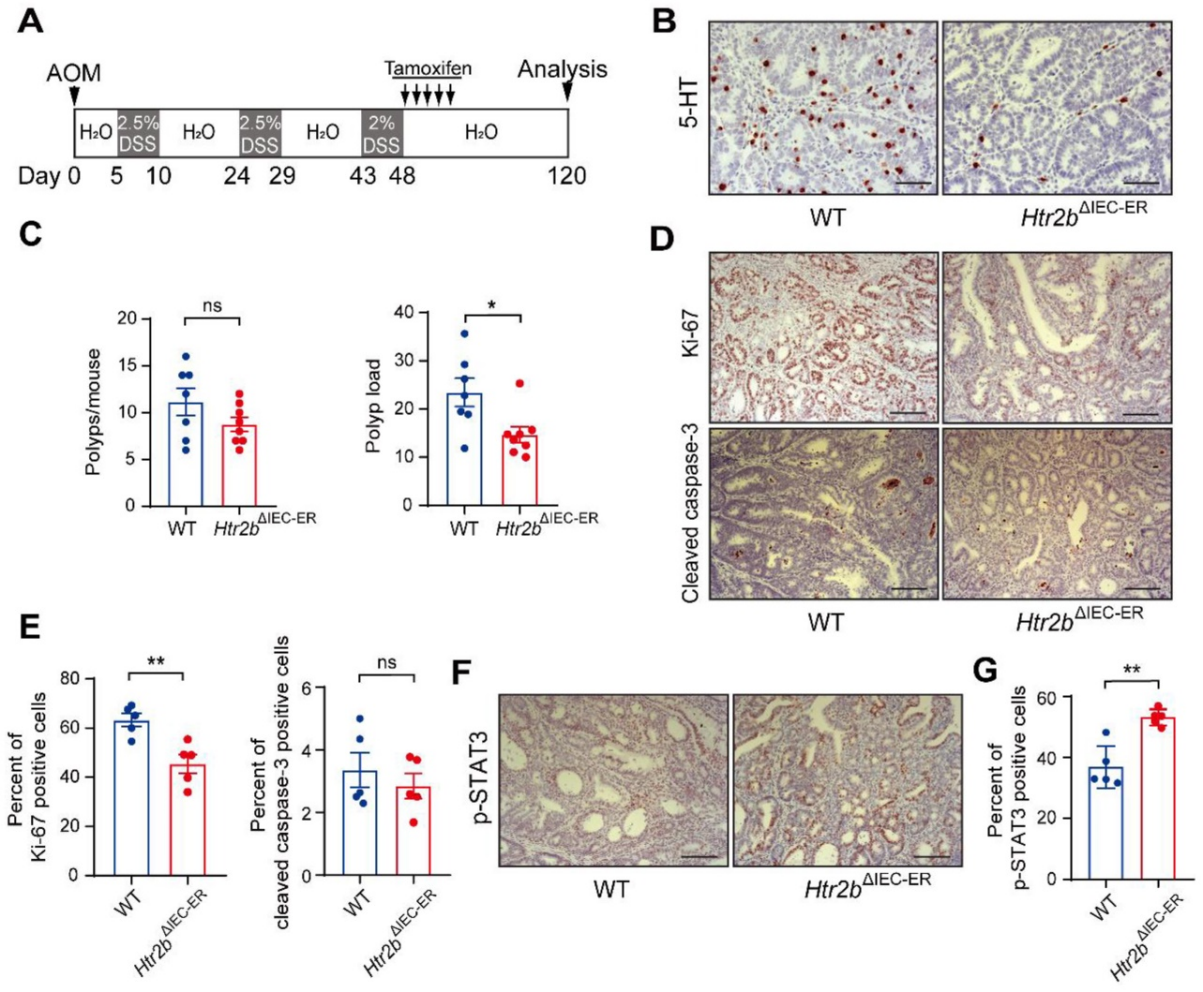
** 5-HT2B promotes tumors in the late stage of CAC.** (**A**) Schematic of inducible *Htr2b* deletion in the AOM/DSS model. *Villin*-CreER^T2^ activation was mediated by tamoxifen injection after the tumors had formed. (**B**) Immunohistochemical analysis of 5-HT in the colonic polyp tissues of WT and *Htr2b*^ΔIEC-ER^ mice. (**C**) The colonic polyp multiplicity and polyp load in WT and *Htr2b*^ΔIEC-ER^ mice given AOM/DSS treatment (n = 7-8). (**D**) Immunohistochemical analysis of Ki-67 and cleaved caspase-3 in colonic polyp tissues of WT and *Htr2b*^ΔIEC-ER^ mice given AOM/DSS treatment. (**E**) The percentage of Ki-67 and cleaved caspase-3 positive cells in colonic polyp tissues of WT and *Htr2b*^ΔIEC-ER^ mice (n = 5). (**F**) Immunohistochemical analysis of p-STAT3 in the colonic polyp tissues of WT and *Htr2b*^ΔIEC-ER^ mice. (**G**) The percentage of p-STAT3-positive cells in colonic polyp tissues of WT and *Htr2b*^ΔIEC-ER^ mice (n = 5). Scale bars, 100 *μ*m. Error bars represent the mean ± SEM. ns: not significant, ^*^P < 0.05, ^**^P < 0.01; unpaired Student's t-test (C, E, G).

Decades of literature have shown that 5-HT promotes tumor proliferation through the activation of its receptors, and that 5-HT-induced cancer cell expansion is closely related to the activities of 5-HT1 and 5-HT2 receptors 15, 38, 39. Therefore, we investigated whether the deletion of 5-HT2B posterior to the tumorigenesis of CAC promotes the aggressiveness of existing tumors. The *Htr2b*^ΔIEC-ER^ mice were treated with tamoxifen on the 48^th^ day after AOM/DSS treatment and *Htr2b* gene was ablated at the “late-stage” of CAC, as illustrated in Figure 5A. The 5-HT level in colonic epithelium cells was significantly reduced in *Htr2b*^ΔIEC-ER^ mice (Figure 5B), which is the same as the previous result in Figure 1D. The polyp load of *Htr2b*^ΔIEC-ER^ mouse decreased significantly, but there was no significant difference in polyp multiplicity (Figure 5C). This result suggests that the deletion of 5-HT2B in the late-stage of CAC does not affect the occurrence of tumors but can inhibit the development of existing tumors. Compared with WT mice, immunohistochemical results showed that tumor proliferation (Ki-67) was significantly decreased in *Htr2b*^ΔIEC-ER^ mice, but there was no difference in apoptosis (cleaved caspase-3) (Figures 5D and E). However, when the *Htr2b* gene was knocked out in mice during the late-stage of CAC, the phosphorylation levels of STAT3 were still increased compared with the WT mice (Figures 5F and G). This result indicates that the reduced tumor aggressiveness observed in *Htr2b*^ΔIEC-ER^ mice is unlikely to be associated with IL-6/STAT3 signaling pathway. Thus, 5-HT/5-HT2B signaling may promote tumor development in the late-stage of CAC through other pathways.

**Figure 6:**
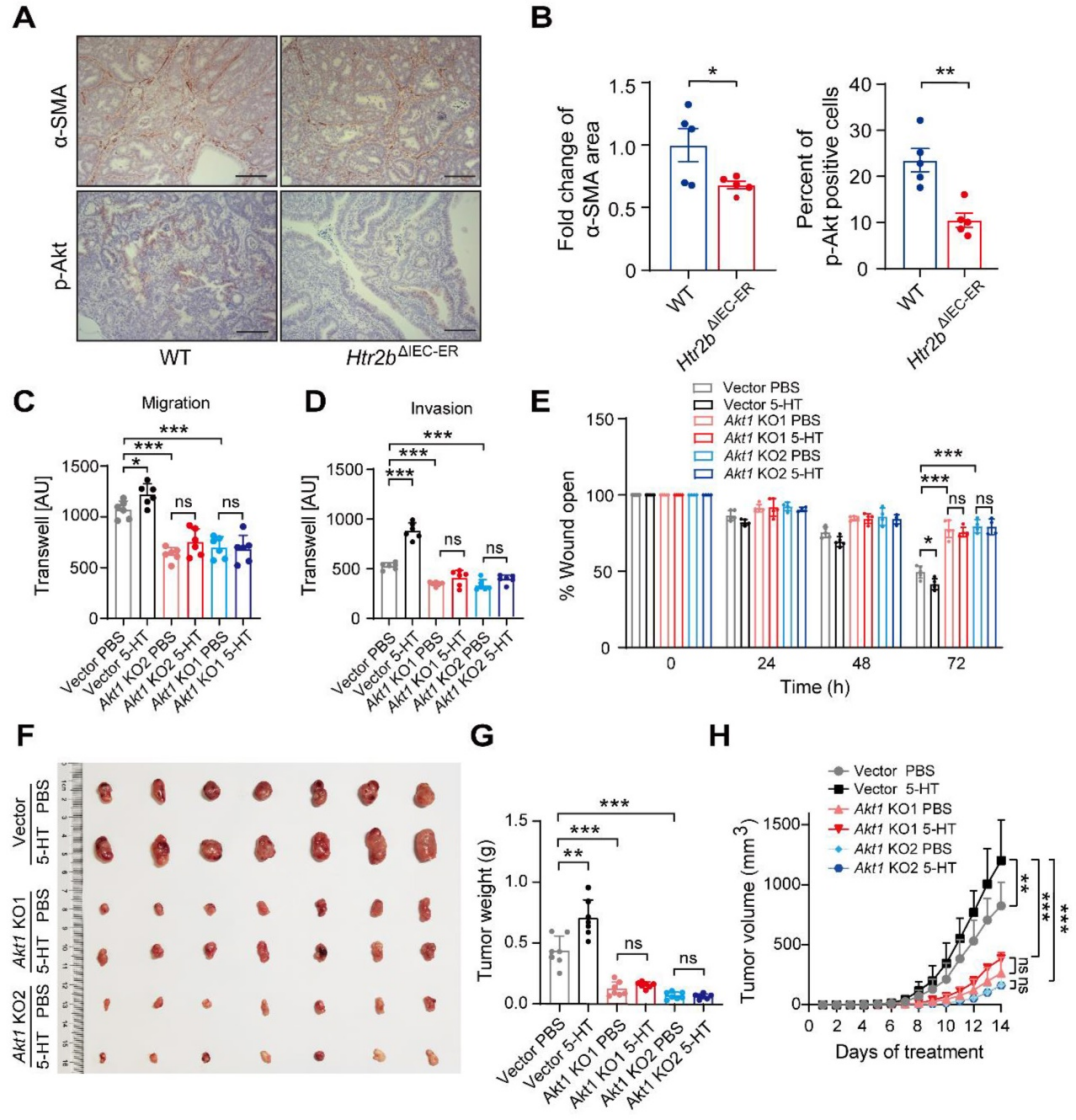
** Knocking out *Akt1* attenuates 5-HT-induced migration and invasion of tumor cells.** (**A**) Immunohistochemical analysis of p-Akt and α-SMA in the colonic polyp tissues of WT and *Htr2b*^ΔIEC-ER^ mice given AOM/DSS treatment. (**B**) Quantification of α-SMA positive area in colonic polyp tissues of WT and *Htr2b*^ΔIEC-ER^ mice (left). The percentage of p-Akt-positive cells in colonic polyp tissues of WT and *Htr2b*^ΔIEC-ER^ mice (n = 5) (right). (**C-D**) Transwell assay results for the migration (**C**) and invasion (**D**) of vector, *Akt1* KO1, and *Akt1* KO2 monoclonal cell lines treated with 5 μM 5-HT or PBS (n = 6). AU: arbitrary unit. (**E**) Wound healing results for the migration of vector, *Akt1* KO1, and *Akt1* KO2 monoclonal cell lines treated with 5 μM 5-HT or PBS (n = 5). (**F-H**) Representative images (**F**), tumor weight (**G**), and tumor volumetric time curve (**H**) of vector, *Akt1* KO1, *Akt1* KO2 allograft tumors seeded in nude mice treated with PBS or 5-HT (n = 7). *Scale bars*, 100 *μm*. Error bars represent the mean ± SEM. ns: not significant, ^*^P < 0.05, ^**^P < 0.01, ^***^P < 0.001; unpaired Student's t-test (B), One-way ANOVA (C-E, G), and Two-way ANOVA (H).

Furthermore, 5-HT signaling significantly impacts angiogenesis 40, an important factor in tumor progression. We confirmed that α-smooth muscle actin (α-SMA, a marker of smooth muscle cell) expression was decreased in *Htr2b*^ΔIEC-ER^ mice (Figures 6A and B), suggesting a decrease in angiogenesis. TGF-β promotes tumor development via Akt phosphorylation 41. Immunohistochemical result showed that the level of phosphorylated Akt was decreased in *Htr2b*^ΔIEC-ER^ mice (Figures 6A and B), suggesting that 5-HT/5HT2B deficiency may reduce tumor development by inhibiting Akt phosphorylation in the late-stage of CAC.

To further explore the role of Akt in 5HT signaling, we used the CRISPR-Cas9 technique to knock out *Akt1* in CT26.WT cells, a mouse colon carcinoma cell line, and screened out two monoclonal cell lines: Akt1 KO1 and Akt1 KO2 (Figure S6A). Western blot results showed that Akt1 protein could not be detected in *Akt1* KO cell lines (Figure S6B). We next analyzed the effect of 5-HT on cell proliferation in Akt1 knockout cell lines. 5-HT can promote the proliferation of tumor cells, which can be attenuated by *Akt1* deletion (Figure S6C). Transwell and wound healing assays indicated that 5-HT could significantly promote the migration and invasion of cells with vector but these promotive effects were not observed in cells with Akt1 knockout (Figures S6D-E and 6B-D). Allograft tumor cells were subcutaneously injected into nude mice, which were then injected intraperitoneally with PBS or 5-HT three times a week to analyze the effect of 5-HT on tumor development after Akt1 knockout. We found that the injection of 5-HT could significantly promote tumor growth, but there was no significant increase in growth with 5-HT treatment in the Akt1 knockout groups (Figures 6F-H). The same results were obtained upon treatment with Akt antagonist GSK690693 (Figures S6F and G). These data suggest that 5-HT can promote tumor growth in the late-stage of the CAC model and that this mechanism is mainly achieved through the effects of Akt1 on the proliferation and migration of tumor cells.

### 5-HT2B is crucial for cancer progression in human colorectal cancer

**Figure 7:**
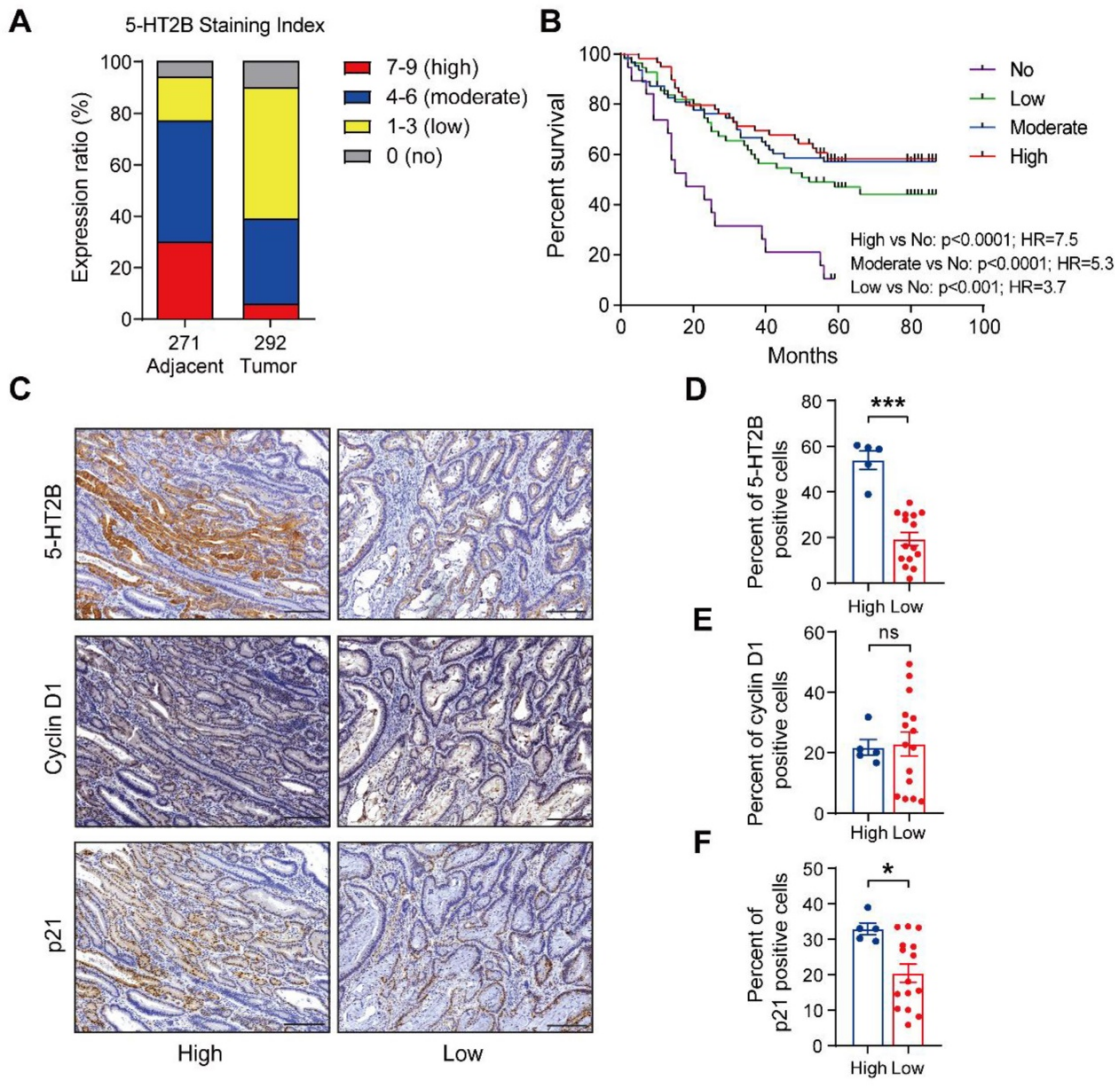
** The expression level of 5-HT2B positively correlates with disease prognosis.** (**A**) Distribution of 5-HT2B expression (high, 7-9; moderate, 4-6; low, 1-3; no, 0) in normal (n = 271) or CRC (n = 292) specimens from humans. (**B**) The 5-year survival rate of CRC patients with different 5-HT2B expression levels in 191 colorectal cancer specimens. (**C**) Immunohistochemical analysis of 5-HT2B, cyclin D1, and p21 in CRC tissue with high or low expression of 5-HT2B. (**D-F**) The positive cell percentages of 5-HT2B (**D**), cyclin D1 (**E**), and p21 (**F**) in CRC tissues with high (n = 5) and low (n = 15) expression of 5-HT2B. Scale bars, 100 *μ*m. Error bars represent the mean ± SEM. ns: not significant, ^*^P < 0.05, ^***^P < 0.001; log-rank test (B) and unpaired Student's t-test (D-F).

To evaluate the clinical relevance of 5-HT2B in human colorectal cancer, we used cBioPortal 42 to mine the Cancer Genome Atlas (TCGA) for 5-HT2B expression levels in 41 adjacent normal colorectal tissue specimens and 287 CRC tissue specimens. Gene expression analysis of TCGA data revealed no significant difference in *Htr2b* expression in the adjacent normal colonic epithelium in comparison to the CRC (Figure S7A). We next examined 5-HT2B protein levels in a tumor tissue microarray (TMA) consisting of 271 adjacent normal colorectal tissue specimens and 292 CRC tissue specimens. An examination of the human cancer specimens showed that approximately 77% of the adjacent normal colorectal specimens exhibited moderate to high 5-HT2B staining, whereas only 39% of the CRC specimens exhibited moderate to high 5-HT2B staining (Figures 7A and S7B). More importantly, overall survival analysis showed that patients with higher, moderate, or lower expression of 5-HT2B in the tumor tissue had a better 5-year survival rate than patients with no positive expression of 5-HT2B (*P* <0.0001, *P* <0.0001 and *P* <0.001, respectively), indicating that 5-HT2B protein level in colorectal tumor cells could serve as a predictor of patient survival. However, there were no significant differences in the survival rate among the patients with higher, moderate, or lower expression of 5-HT2B (Figure 7B). It has been suggested that the expression of the 5-HT2B protein may play a protective role in colorectal cancer. This effect depends only on whether 5-HT2B is expressed but is irrelevant to the expression level of 5-HT2B. To evaluate the clinical relevance of the relationship between 5-HT2B expression and TGF-β signaling activity, we detected 5-HT2B, cyclin D1, and p21 expression in a CRC tissue microarray using immunohistochemistry. We found that in a cohort of 20 patients with CRC, both the adjacent normal tissues and tumor tissues with high expression of 5-HT2B showed high expression of p21 and that the tissues with low expression of 5-HT2B showed the opposite expression patterns; however, there was no significant correlation between 5-HT2B and cyclin D1 expression (Figures 7C-F and S7C). Taken together, these data suggest that the protein expression of 5-HT2B positively correlates with disease prognosis and illustrates significant genetic cooperation among 5-HT, 5-HT2B, and TGF-β signaling in human CRC.

## Discussion

**Figure 8:**
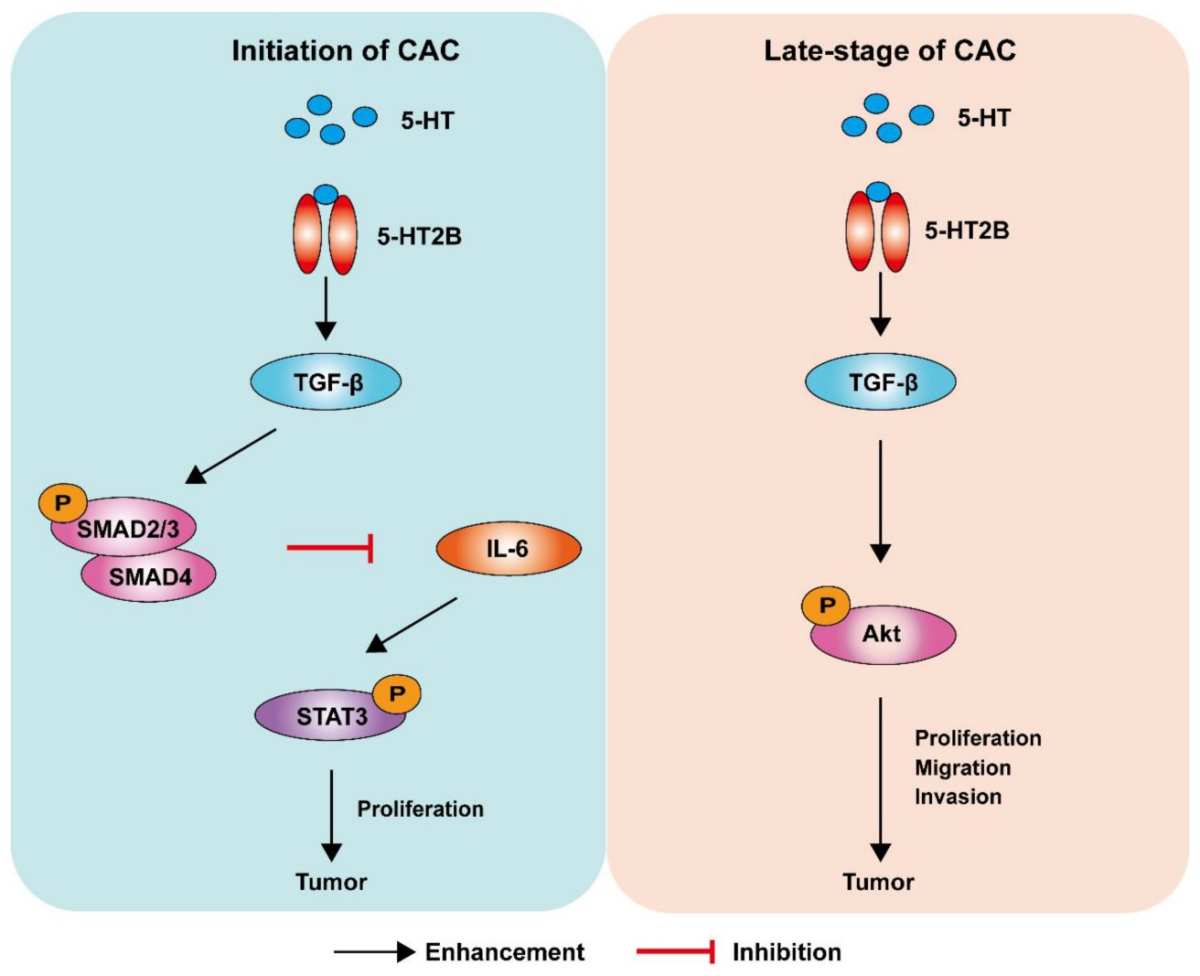
** Model depicting a mechanism by which 5-HT regulates CAC tumorigenesis and development.** Left: In premalignant cells, 5HT/5-HT2B enhances SMAD2/3 phosphorylation and SMAD4 expression via TGF-β, which inhibits the IL-6/STAT3 signaling pathway and suppresses the proliferation of tumor cells. Right: In tumor cells, 5HT/5-HT2B enhances Akt phosphorylation through TGF-β, which promotes the proliferation, migration, and invasion of tumor cells.

For decades, the causal relationship between chronic inflammation and cancer development has been recognized 28. CAC acts as a tumor that is closely related to early inflammation. Here, we demonstrated that specific 5-HT2B ablation in IECs interfered with tumor formation and growth in a mouse model of CAC. We confirmed that 5-HT2B deficiency in IECs significantly attenuated the effects of 5-HT on inflammation and tumors in the epithelium, suggesting that 5-HT2B plays an important role in the regulation of CAC by 5-HT. Our study revealed that 5-HT/5HT2B suppressed CAC tumorigenesis by regulating canonical TGF-β signaling, which protects against epithelial damage and inflammation. However, once CAC tumors were formed, 5-HT facilitated CAC progression by promoting proliferation, migration, and invasion, depending on the activation of a noncanonical TGF-β effector (Figure 8). 5-HT may function as a context-dependent tumor-suppressor or a factor with oncogenic functions, which is realized by 5-HT/5-HT2B/TGF-β axis in CAC.

As a classical inflammatory factor, IL-6 is also involved in the regulation of inflammation and cancer. In agreement with previous evidence for the role of IL-6 in promoting colorectal tumorigenesis in CAC mouse models 28, 31, we found that DSS-treated *Htr2b*^ΔIEC^ mice produced increased levels of IL-6 and that IL-6 regulated the survival and proliferation of IECs and their preneoplastic derivatives during acute colitis and CAC induction. The tumor-promoting effects of IL-6 are mainly dependent on the activation of the transcription factor STAT3 31. STAT3 is an important regulator of colon tumor cell survival and proliferation 28, 31. Inhibition of STAT3 or IL-6 greatly reduces the tumor burden in inflammation-associated colon cancer 31. Consistent with these results, STAT3 activation was increased in *Htr2b*^ΔIEC^ mice with DSS-induced colitis. Ablation of STAT3 expression in the IECs of *Htr2b*^ΔIEC^ mice effectively inhibited the induction and growth of CAC, demonstrating that IL-6/STAT3 plays a critical role in the regulation of CAC by 5-HT/5HT2B.

Inflammation is a complex biological immune response that can be caused by tissue damage induced by pathogens or irritants. Mounting evidence indicates that within the intestinal mucosal layer, 5-HT can act as a proinflammatory or anti-inflammatory signaling molecule 43. Khan and colleagues demonstrated that mice were protected from colitis when mucosal 5-HT synthesis was diminished by the TPH inhibitor para-chlorophenylalanine or when *Tph1* was knocked out in mice 12. By contrast, another study using a mouse model of colitis showed that loss of SERT increased the 5-HT level and reduced the severity of inflammation in the colon 44. Similarly, we found that mice with 5-HT2B deletion had a higher histological damage score than WT littermates in the CAC model and showed more body weight loss in the DSS-induced colitis model. Furthermore, treatment with the 5-HT2B antagonist SB-204741 or Compound-15 resulted in increased body weight loss in DSS-induced colitis model mice. In contrast, fluoxetine, an agonist of 5-HT2B, resulted in a higher weight than the control treatment in DSS-induced colitis mice. Collectively, these studies contribute new knowledge regarding the protective actions of 5-HT2B activation and provide evidence of an anti-inflammatory role for 5-HT2B in the initial stage of the CAC model.

Here, we revealed that TGF-β1 expression was markedly downregulated in *Htr2b*^ΔIEC^ mouse models of colitis and CAC. Correspondingly, we observed that the levels of phosphorylated SMAD2/3 were decreased in* Htr2b*^ΔIEC^ mice. In precancerous cells, TGF-β is mainly a tumor inhibitor that depresses cell proliferation or induces apoptosis. However, in the late-stage of cancer progression, TGF-β acts as a metastasis promoter by inducing EMT, to increase the invasiveness of cancer cells, and by inducing the expression of genes that promote metastasis to and colonization of secondary organs 45. TGF-β signaling regulates the activation of downstream SMAD and non-SMAD pathways 46. In the canonical SMAD-dependent signaling pathway, TGF-β binds to its receptors on the cell membrane and induces a signaling cascade by phosphorylating SMAD2 and SMAD3. Activated SMAD2 and SMAD3 form a SMAD complex with SMAD4 and translocate into the nucleus. In the nucleus, the SMAD complex binds to the promoter regions of TGF-β target genes and regulates the transcription of these genes 5, 46, 47. Our results suggest that 5-HT inhibits the occurrence of CAC by activating the SMAD-dependent TGF-β signaling pathway rather than SMAD-independent signaling in the initial stage of CAC.

Further, TGF-β is related to the progression of late-stage tumors depending upon the activation of the SMAD-independent TGF-β signaling pathway, including mitogen-activated protein (MAP) kinases, INK, PI3 K-Akt, and small GTPases 46. SMAD-independent pathways also induce a proangiogenic environment and stimulate tumor angiogenesis, and increased TGF-β expression has been linked to an increase in microvessel density in a few tumor types, which is related to a poor prognosis 45, 46. TGF-β is considered to be an inducer of EMT, and several studies have confirmed the crucial roles for TGF-β-induced EMT in tumor progression 8. Our results support the previous conclusion, as the expression levels of α-SMA and phosphorylated Akt decreased in *Htr2b*^ΔIEC-ER^ mice, revealing a decrease in angiogenesis and indicating that the inhibition of Akt-mediated EMT reduced the proliferation, migration, and invasion of CAC cells. Our results indicate that 5-HT/5-HT2B promotes tumors reliant on SMAD-independent TGF-β signaling in the late-stage of CAC.

However, this conclusion was obtained in *Htr2b*^ΔIEC-ER^ mice with ablation of 5-HT2B in the late-stage of CAC. For studies in humans, we could not obtain clinical samples of established tumors with deleted 5-HT2B expression. However, compared with the *Htr2b*^ΔIEC-ER^ mice model, humans are more similar to the *Htr2b*^ΔIEC^ mice model. The human TMA showed that patients without 5-HT2B detection in tumors had a lower survival rate (Figure 7B). This corresponds to the result that *Htr2b*^ΔIEC^ mice showed more severe tumors (Figure 1B). It seems that the patients' survival rate depended on the expression rather than the abundance of 5-HT2B. These mechanisms need to be further studied.

Finally, it should be noted that most of our conclusions were obtained from AOM/DSS induced CAC model in mice. Whether these conclusions are still applicable to colitis-independent tumors (such as spontaneous colon cancer) is unclear. This is the limitation of our research, which needs to be further studied.

## Conclusion

In summary, our work elucidates that 5-HT functions as a critical tumor suppressor during CAC initiation via 5-HT2B but promotes tumor progression in the late-stage of CAC (Figure 8). Although 5-HT/5-HT2B/TGF-β signaling could act protectively against early carcinogenic events in the colon, the same system could promote the proliferation, migration, and invasion of established colorectal tumors in the late-stage of CAC. Our findings provide a new understanding of the role of 5-HT in the initiation and progression of CAC, offering a new angle of view for the long-standing debate on whether the 5-HT signal promotes or inhibits tumors.

## Supplementary Material

Supplementary figures and table.

## Abbreviations

5-HT: 5-Hydroxytryptamine
5-HT2B: 5-Hydroxytryptamine receptor 2B
α-SMA: α-Smooth muscle actin
ANOVA: Analysis of variance
AOM: Azoxymethane
CAC: Colitis-associated cancer
CRC: Colorectal cancer
DSS: Dextran sodium sulfate
EC: Enterochromaffin
EMT: Epithelial-mesenchymal transition
ERK: Mitogen-activated protein kinase 1
FBS: Fetal bovine serum
H&E: Hematoxylin and eosin
IBD: Inflammatory bowel diseases
IECs: Intestinal epithelial cells
IL-6: Interleukin-6
MAP: Mitogen-activated protein
PBS: Phosphate buffer saline
qPCR: Quantitative Real-time PCR
SERT: Serotonin transporter
SMAD: Small mothers against decapentaplegic
STAT3: Signal transducer and activator of transcription-3
STR: Short tandem repeat
TCGA: The cancer genome atlas
TGF-β: Transforming growth factor β
TMA: Tumor tissue microarray
TPH1: Tryptophan hydroxylase I
WT: Wild-type
