# Supplementary material for: 5-HT2B-mediated serotonin activation in enterocytes suppresses colitis-associated cancer initiation and promotes cancer progression
Source: Theranostics. 2022 May 9;12(8):3928–45. doi: 10.7150/thno.70762 (PMC9131283; doi:10.7150/thno.70762)
Supplement: Supplementary file 1 — Supplementary figures and table. [file thnov12p3928s1.pdf]

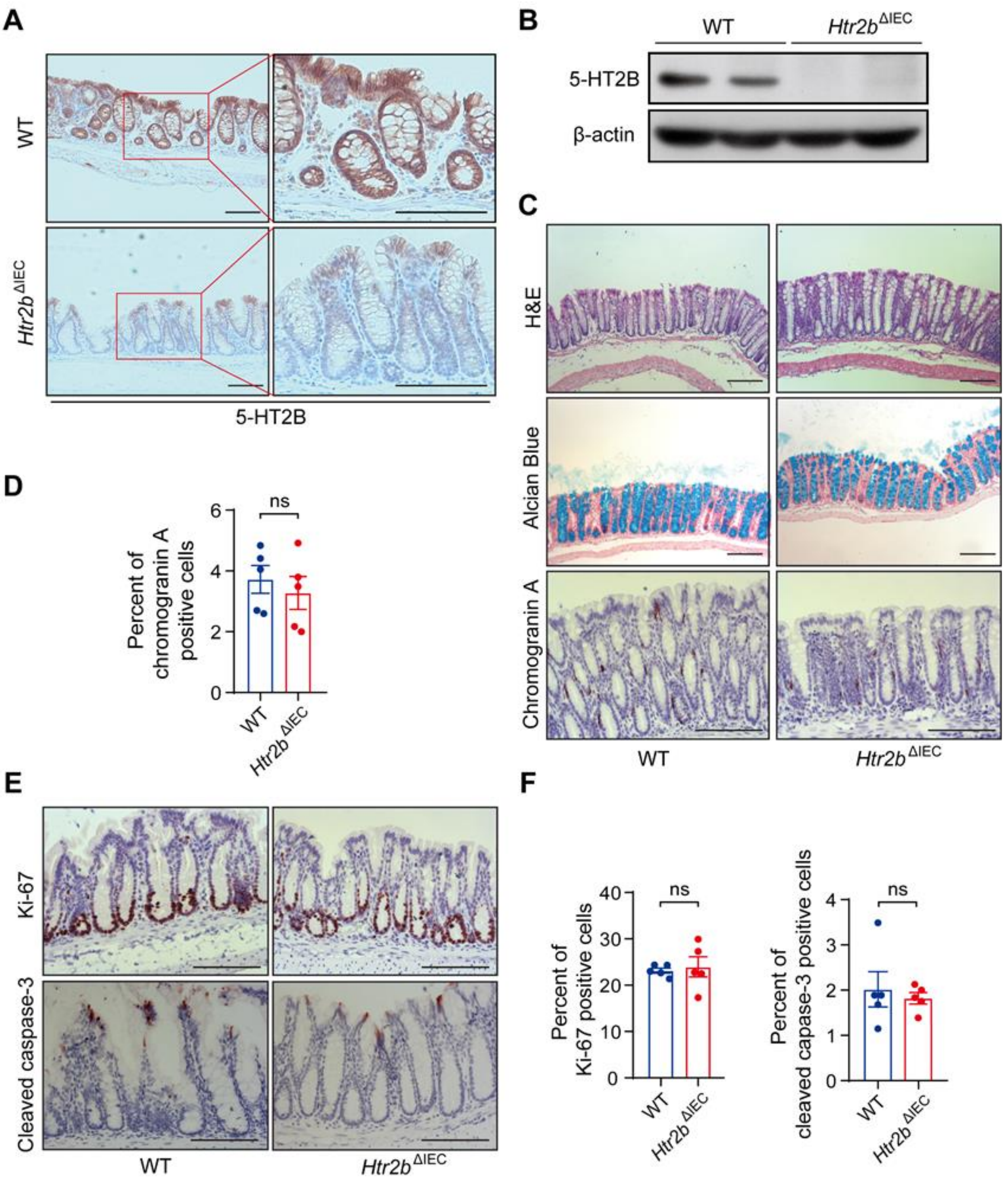

Figure S1. 5-HT2B is dispensable in the intestinal epithelium. (**A**) Immunohistochemical staining for 5-HT2B in the colonic epithelium of WT and *Htr2b*$^{\Delta IEC}$ mice. (**B**) 5-HT2B expression in the colonic epithelium of WT and *Htr2b*$^{\Delta IEC}$ mice. Each lane represents a different mouse. Data are representative of three independent experiments. (**C**) Morphological changes in the intestinal epithelium in WT and *Htr2b*$^{\Delta IEC}$ mice. H&E-stained sections of control and experimental colon tissues revealed no gross changes in architecture or histology. Goblet cells were marked

by Alcian Blue staining, and immunohistochemical analysis against Chromogranin A was used to mark enteroendocrine cells. These cells appeared to be properly differentiated in the intestines of *Htr2b*$^{\Delta IEC}$ mice. (**D**) The percentage of Chromogranin A positive cells (positive epithelial cells/total epithelial cells) in the colonic epithelium of WT and *Htr2b*$^{\Delta IEC}$ mice (n = 5). (**E**) Immunohistochemical staining for Ki-67 and cleaved caspase-3 in the colonic epithelium of WT and *Htr2b*$^{\Delta IEC}$ mice. (**F**) The percentage of Ki-67 and Cleaved caspase-3 positive cells (positive epithelial cells/total epithelial cells) in the colonic epithelium of WT and *Htr2b*$^{\Delta IEC}$ mice (n = 5). Scale bars, 100 μm. Error bars represent the mean ± SEM. ns: not significant; unpaired Student's t-test (D, F).

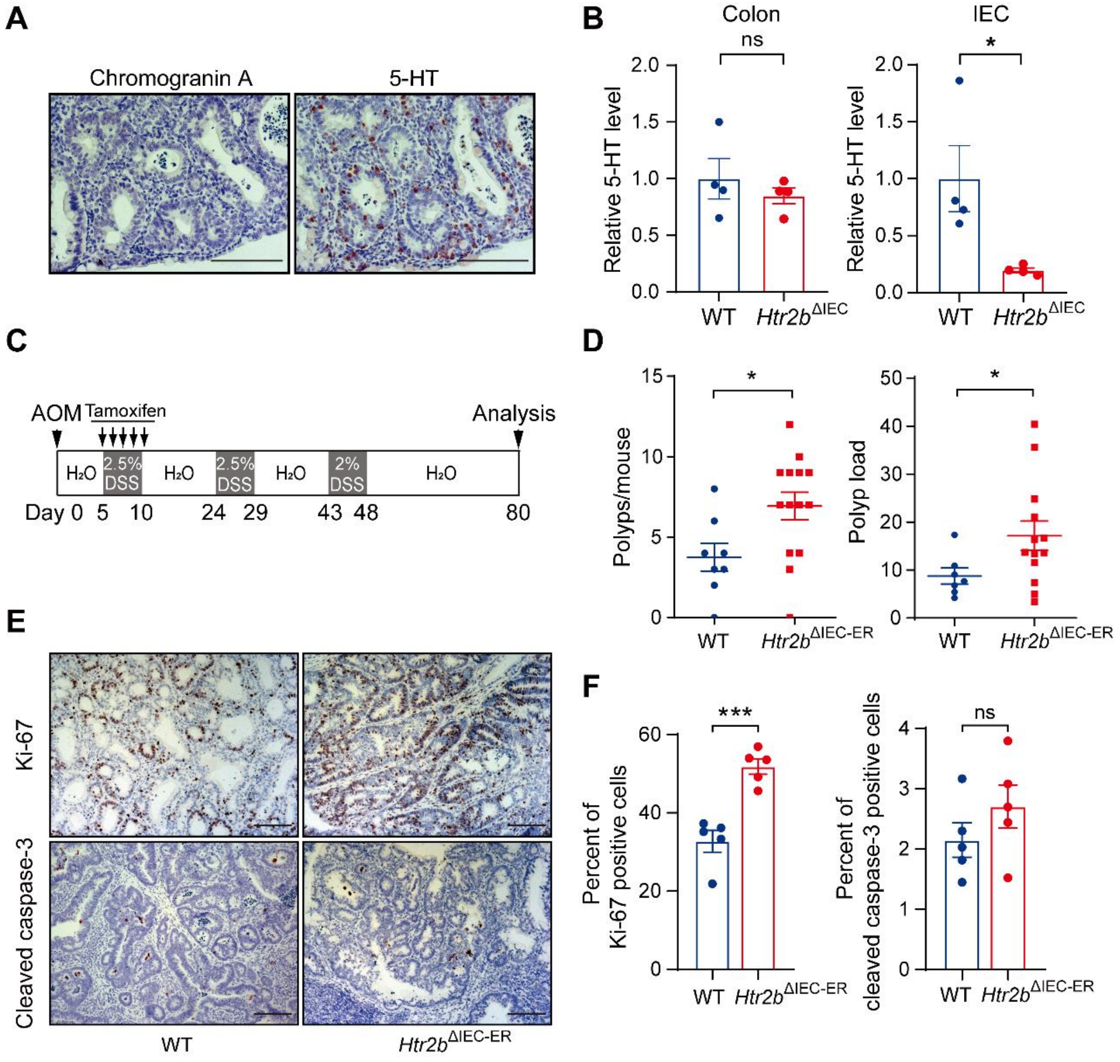

Figure S2. 5-HT2B knockout aggravates DSS-induced CAC in the precancerous stage. (**A**) Immunohistochemical analysis of chromogranin A and 5-HT in the colonic polyp tissues of WT mice. (**B**) 5-HT values in colonic tissues and IECs of WT and *Htr2b*$^{\Delta IEC}$ mice determined by HPLC. (**C**) Schematic of inducible *Htr2b* deletion in the AOM/DSS model. *Villin*-CreERT2 activation was mediated by tamoxifen injection before tumors developed. (**D**) The intestinal polyp multiplicity and polyp load of WT (n = 7) and *Htr2b*$^{\Delta IEC\text{-}ER}$ (n = 13) mice following the induction of CAC using AOM/DSS. (**E**) Immunohistochemical analysis of Ki-67 and cleaved caspase-3 in colonic polyp tissues of WT and *Htr2b*$^{\Delta IEC\text{-}ER}$ mice. (**F**) The percentage of Ki-67 and cleaved caspase-3 positive cells in colonic polyp tissues of AOM/DSS-treated WT and *Htr2b*$^{\Delta IEC\text{-}ER}$ mice

(n = 5). Scale bars, 100 μm. Error bars represent the mean ± SEM. Each data point represents a single mouse. ns: not significant, $^{*}P < 0.05$, $^{***}P < 0.001$; unpaired Student's t-test (B, D, F).

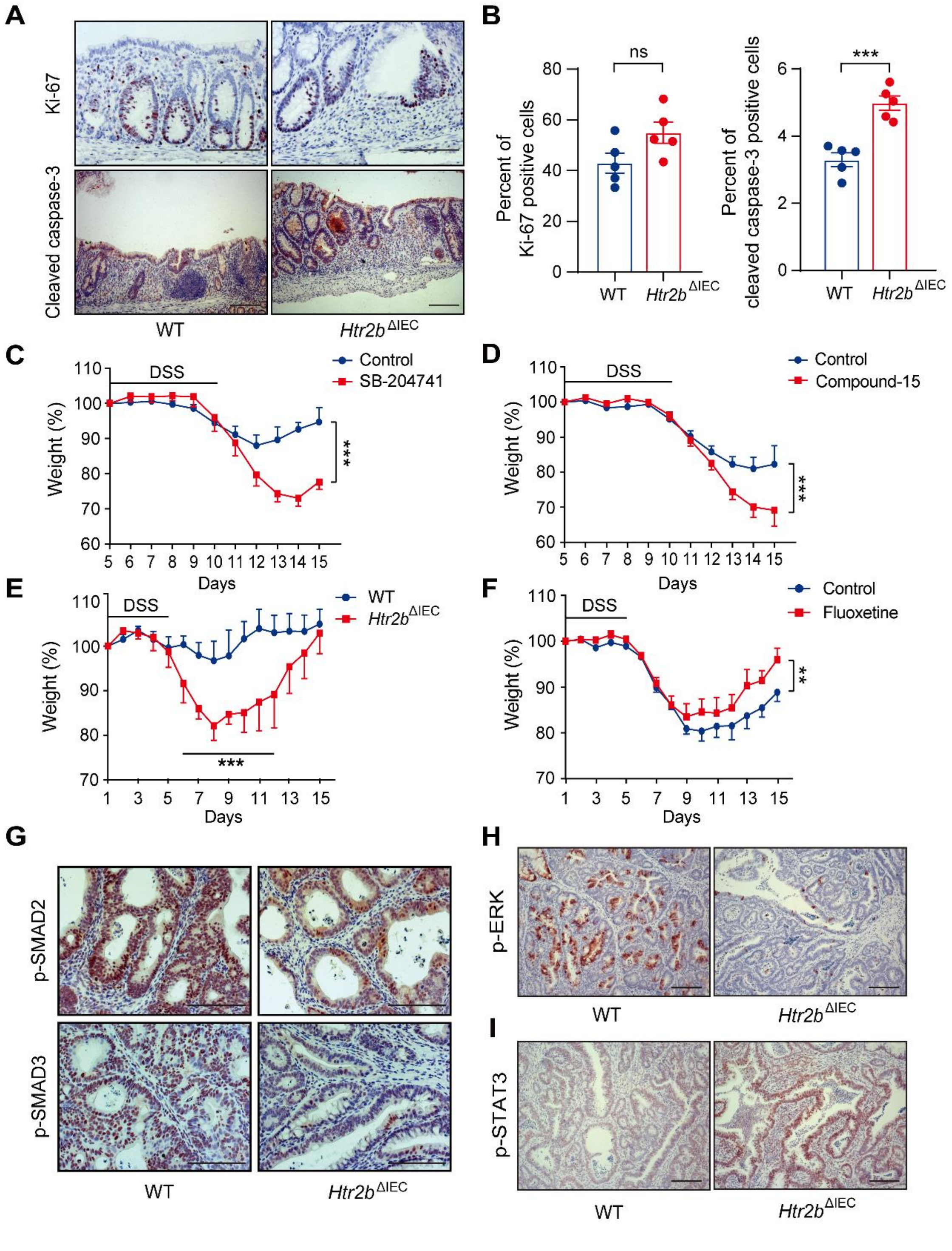

Figure S3. The deficiency of 5-HT2B increases the severity of colitis. (**A**) Immunohistochemical staining for Ki-67 and cleaved caspase-3 in the colonic epithelium of WT and *Htr2b*$^{\Delta IEC}$ mice on day 15. (**B**) The percentage of Ki-67 and cleaved caspase-3 positive cells in AOM/DSS-treated WT and *Htr2b*$^{\Delta IEC}$ mice (n = 5). (**C-D**) The effects of the 5-HT2B antagonists SB-204741 (**C**) (n = 9) and Compound-

15 (**D**) (n = 9) on the change in total body weight of mice induced by AOM/DSS. (**E**) The effect of *Htr2b* knockout on the change in total body weight induced by DSS (n = 8). (**F**) The effect of the 5-HT2B agonist fluoxetine on the change in total body weight induced by DSS (n = 9). (**G-I**) Immunohistochemical analysis of p-SMAD2, p-SMAD3 (**G**), p-ERK (**H**), and p-STAT3 (**I**) in colonic polyp tissues of WT and *Htr2b*$^{\Delta IEC}$ mice on day 80 after induction by AOM/DSS. Scale bars, 100 μm. Error bars represent the mean ± SEM. ns: not significant, $^{**}P < 0.01$, $^{***}P < 0.001$ between the indicated treatments on the indicated days (E); unpaired Student's t-test (B), Two-way ANOVA (C-F).

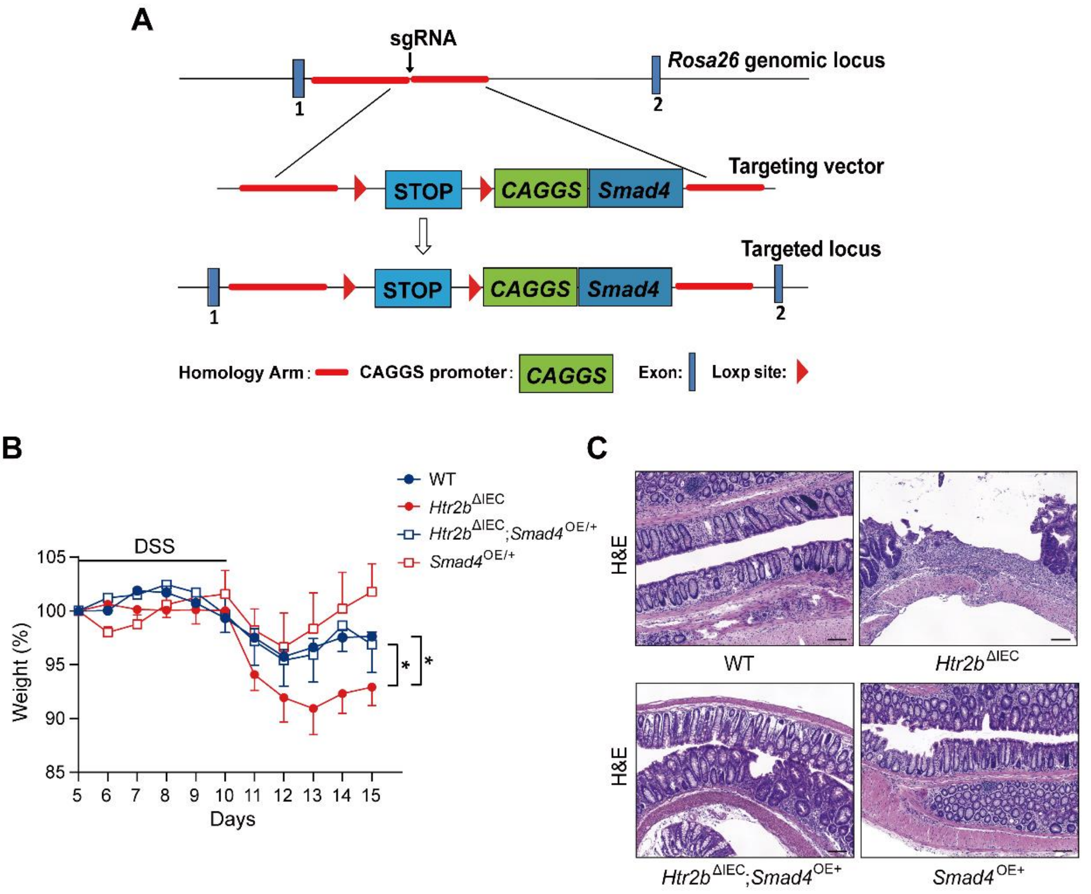

Figure S4. Overexpression of SMAD4 alleviates colitis caused by 5-HT2B deletion. (**A**) Schematic diagram of the targeting strategy for the generation of mice with inducible *Smad4* overexpression using the CRISPR/Cas9 method. (**B**) Body weight of WT, *Htr2b*$^{\triangle IEC}$, *Htr2b*$^{\triangle IEC}$; *Smad4*$^{OE+}$ and *Smad4*$^{OE+}$ mice during AOM/DSS treatment (n = 5). (**C**) H&E staining of colon sections from WT, *Htr2b*$^{\triangle IEC}$, *Htr2b*$^{\triangle IEC}$; *Smad4*$^{OE+}$ and *Smad4*$^{OE+}$ mice with acute colitis on day 15. Scale bars, 100 μm. Error bars represent the mean ± SEM. $^{*}P < 0.05$; Two-way ANOVA (B).

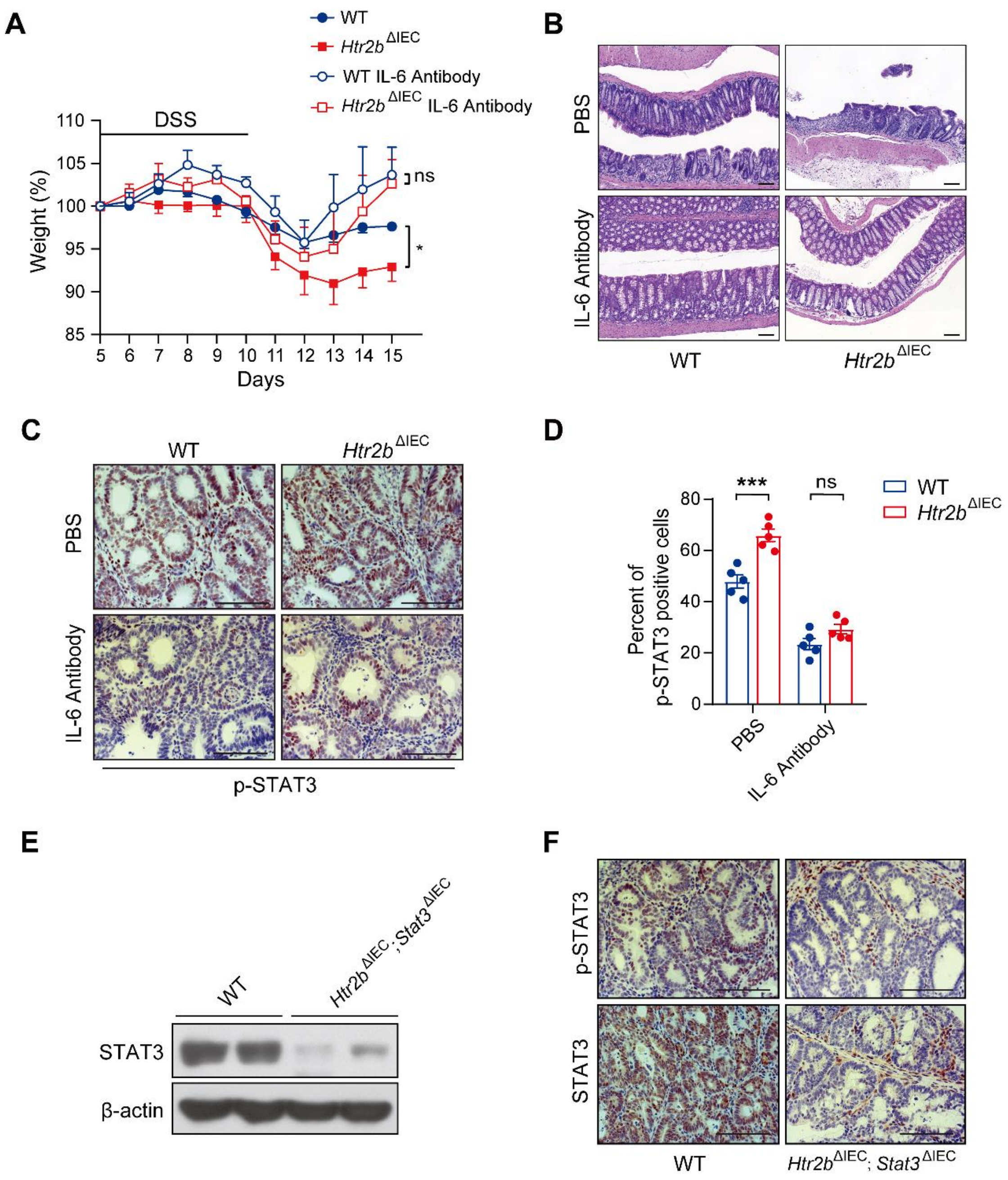

Figure S5. Effects of IL-6 antibody treatment on colitis and CAC. (**A**) Body weight of WT and *Htr2b*$^{\Delta IEC}$ mice treated with IL-6 antibody or PBS during AOM/DSS treatment (n = 5). (**B**) H&E staining of colon sections from WT and *Htr2b*$^{\Delta IEC}$ mice treated with IL-6 antibody or PBS on day 15 of AOM/DSS treatment. (**C**) Immunohistochemical analysis of p-STAT3 in colonic polyp tissues from WT and *Htr2b*$^{\Delta IEC}$ mice treated with PBS or IL-6 antibody. (**D**) The percentage of p-STAT3-positive cells in colonic polyp tissues of WT and *Htr2b*$^{\Delta IEC}$ mice treated with PBS or

an IL-6 antibody (n = 5). (**E**) The expression of STAT3 in the colonic epithelium of WT and *Htr2b*$^{\Delta IEC}$; *Stat3*$^{\Delta IEC}$ mice. Each lane represents a different mouse. (**F**) Immunohistochemical staining for p-STAT3 and STAT3 in the colonic polyp tissues of WT and *Htr2b*$^{\Delta IEC}$; *Stat3*$^{\Delta IEC}$ mice. Scale bars, 100 μm. Error bars represent the mean ± SEM. ns: not significant, $^{*}P < 0.05$, $^{***}P < 0.001$; Two-way ANOVA (A, D).

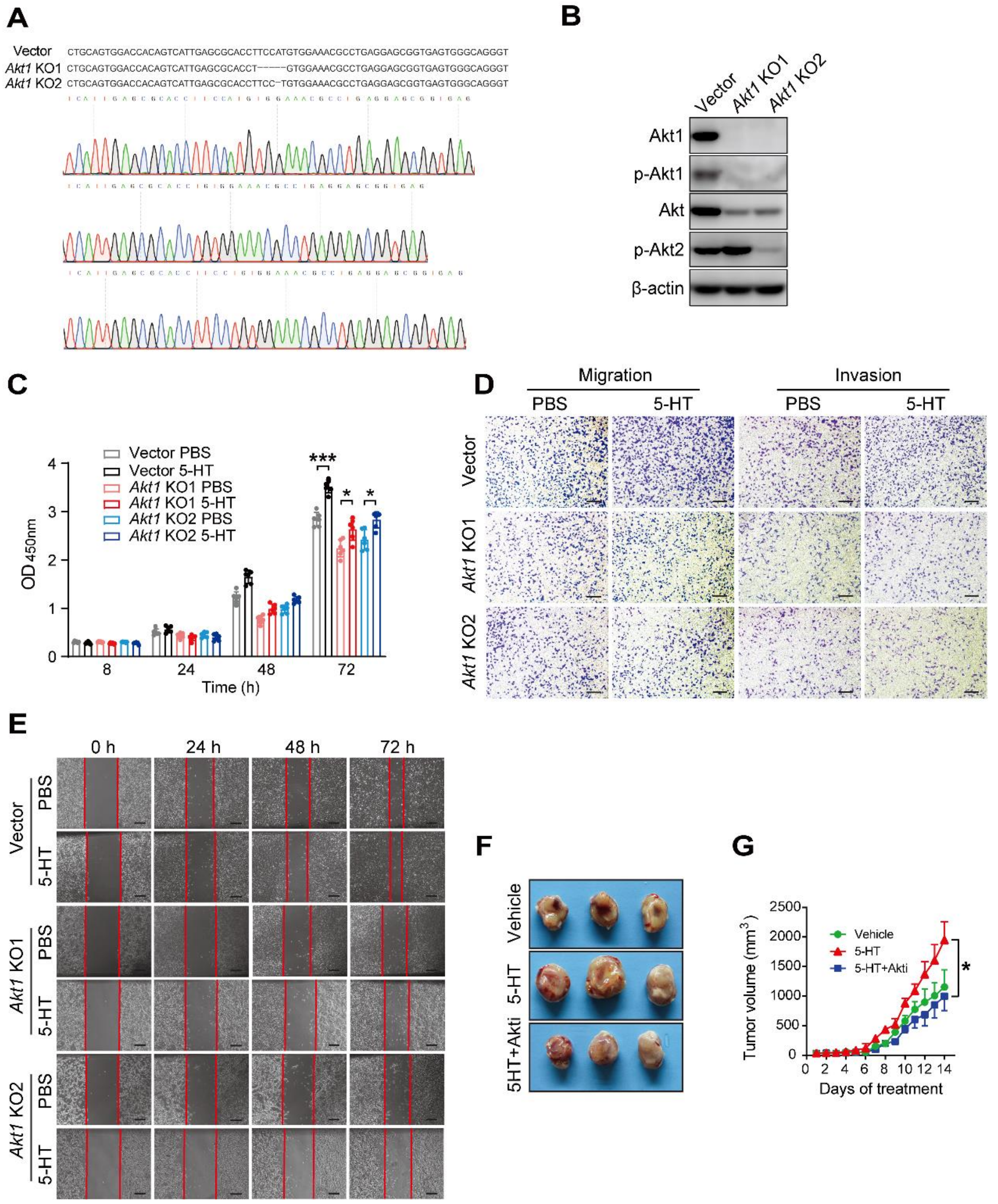

Figure S6. 5-HT/Akt1 promote tumor in vivo. (**A**) KO strategy and sequencing of vector, *Akt1* KO1, and *Akt1* KO2 cell lines generated using the CRISPR-Cas9 technique. (**B**) The expression levels of Akt1, p-Akt1, Akt, and p-Akt2 in vector, *Akt1* KO1, and *Akt1* KO2 cell lines. (**C**) CCK-8 assay results ($OD_{450\ nm}$ readings) of vector, *Akt1* KO1, and *Akt1* KO2 cell lines treated with 5 μM 5-HT or PBS (n = 6). (**D-E**) Representative images of Transwell assay (**D**) and wound healing (**E**). (**F-G**) Representative images (**F**) and tumor volume curves (**G**) of allograft tumors seeded in nude mice and treated

with vehicle, 5-HT, or 5 - HT + Akti (GSK-690693). Scale bars, 100 μm. Error bars represent the mean ± SEM. $^{*}P < 0.05$, $^{***}P < 0.001$; One-way ANOVA (C) and Two-way ANOVA (G).

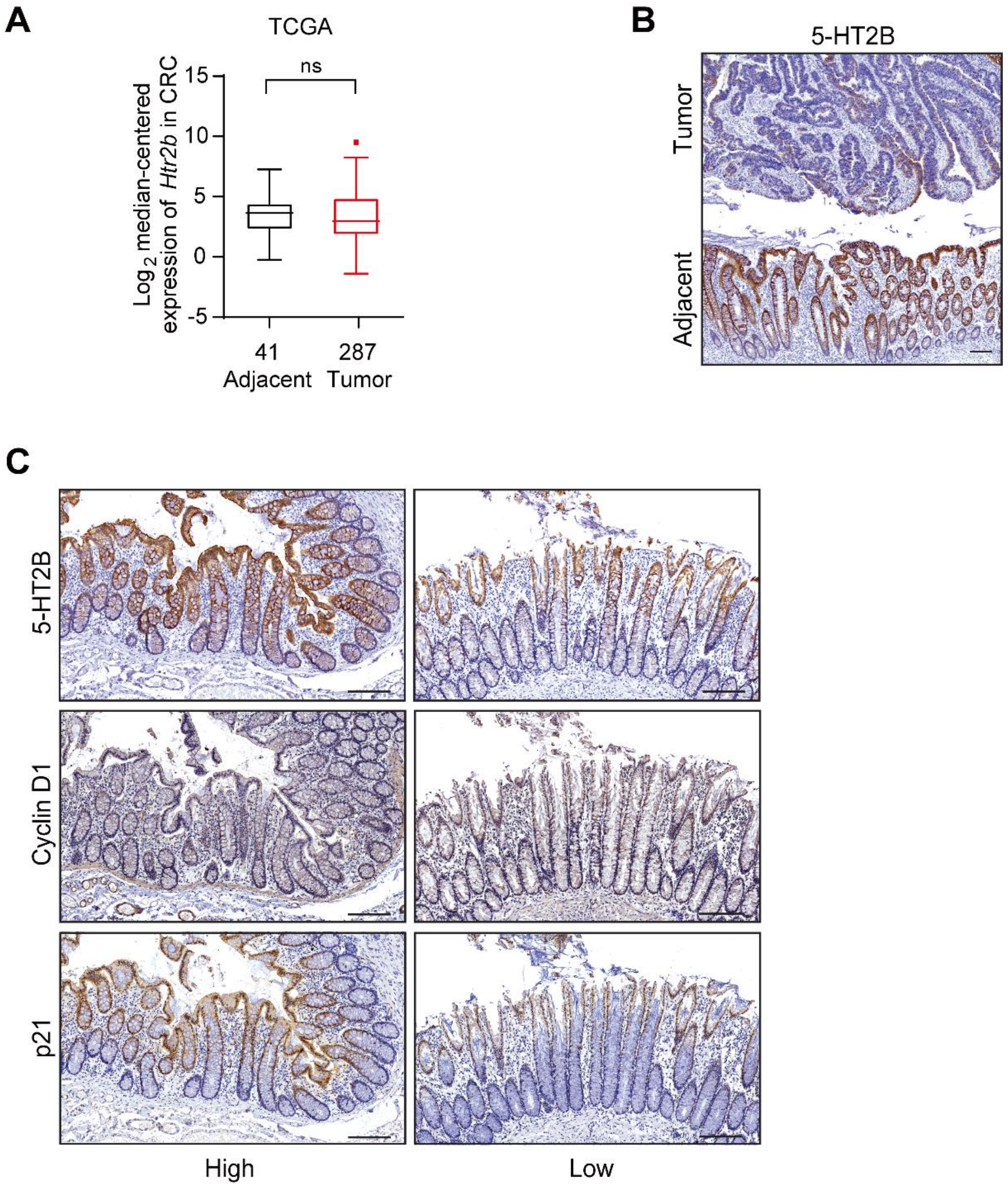

Figure S7. 5-HT2B is expressed in CRC patient tumor samples. (**A**) Analysis of Htr2b expression levels in human colon adjacent tissue (n = 41) and cancer tissue (n = 287) using online TCGA microarray data. All the results are shown as box plots representing the median, 25th, and 75th percentiles as boxes. The 75th percentile plus 1.5 times IQR and the 25th percentile minus 1.5 times IQR as bars. Values that are greater than those bars as individual points. TCGA: The Cancer Genome Altas. (**B**) Immunohistochemical analysis of 5-HT2B in CRC tissue. (**C**) Immunohistochemical analysis of 5-HT2B, cyclin D1, and p21 in CRC adjacent tissue with high or low

expression of 5-HT2B. Scale bars, 100 μm. Error bars represent the mean ± SEM. ns: not significant; unpaired Student's t-test (A).

Table S1.

Primers used for quantitative RT-PCR

Target Gene	Forward Primer (5′-3′)	Reverse Primer (5′-3′)
IL-6	TTGGGACTGATGCTGGTGAC	TTGCCATTGCACAACTCTTTTC
TNF-α	TCTGTGAAGGGAATGGGTGTT	CAGGTCACTGTCCCAGCATC
IFN-γ	CGCCATCAGGGCAGATCTAA	ACCGACTCCTTTTCCGCTTC
IL-1β	TGGGAAACAACAGTGGTCAGG	CCATCAGAGGCAAGGAGGAA
KC	GCACCCAAACCGAAGTCATAG	AGAAGCCAGCGTTCACCAGA
MIP-2	GCAAGGCTAACTGACCTGGAA	CAACATCTGGGCAATGGAAT
TGF-β1	ACTGGAGTTGTACGGCAGTG	GGGGCTGATCCCGTTGATTT
GAPDH	ATGACATCAAGAAGGTGGTGA	TCCTTGGAGGCCATGTAGG
